# A review of electro-hydrogen synergistic systems: from key material breakthroughs, multi-timescale control to full-chain integration

**DOI:** 10.1039/d6ra02235a

**Published:** 2026-07-13

**Authors:** Jie Wang, Bing Hu, Lijun Xu, Xiaochao Fan, Jiading Jiang

**Affiliations:** a Xinjiang Key Laboratory of Green Hydrogen Production, Storage and Use Technology, Department of Energy Engineering, Xinjiang Institute of Engineering Urumqi Xinjiang 830023 China hb05@xjie.edu.cn

## Abstract

Under the carbon peaking and carbon neutrality targets, electro-hydrogen synergetic systems have emerged as a promising pathway for cross-temporal energy conversion and deep decarbonization across power, transport, and industrial sectors *via* the electricity-hydrogen-electricity. The system can enhance renewable energy integration, improve grid resilience, and support low-carbon transitions in hard-to-abate sectors. However, their large-scale deployment is still constrained by bottlenecks in key materials, efficiency coordination, dynamic matching, safety, and techno-economic performance. The review develops a framework spanning materials, components, systems, and market applications, with a focus on key material innovation, multi-timescale regulation, and full-chain integration. First, recent advances and critical challenges in water electrolysis technologies, including PEM, ALK, AEM, and SOEC, are reviewed in terms of catalyst materials, membrane electrode structures, stack-level *in situ* diagnostics, and durability under fluctuating operating conditions. Second, high-pressure gaseous, cryogenic liquid, and solid-state hydrogen storage pathways are comparatively assessed, revealing trade-offs among energy density, efficiency loss, safety, cost, and infrastructure compatibility. Third, the review summarizes coupling architectures for electricity-hydrogen-heat-gas multi-energy systems, as well as dynamic response control and multi-timescale optimal scheduling from microgrids and industrial parks to regional integrated energy systems. Finally, the application potential of electro-hydrogen synergetic systems in renewable energy consumption, grid ancillary services, and industrial decarbonization is discussed, and key scientific questions and suggestions for large-scale demonstration and commercialization are proposed.

## Abstract 2

Electro-hydro synergy explored *via* material innovation, dynamic optimization, and utilization. Multi-time-scale control framework is constructed. Application value and key questions are analyzed. Suggestions are proposed for decarbonization.

## Graphical abstract

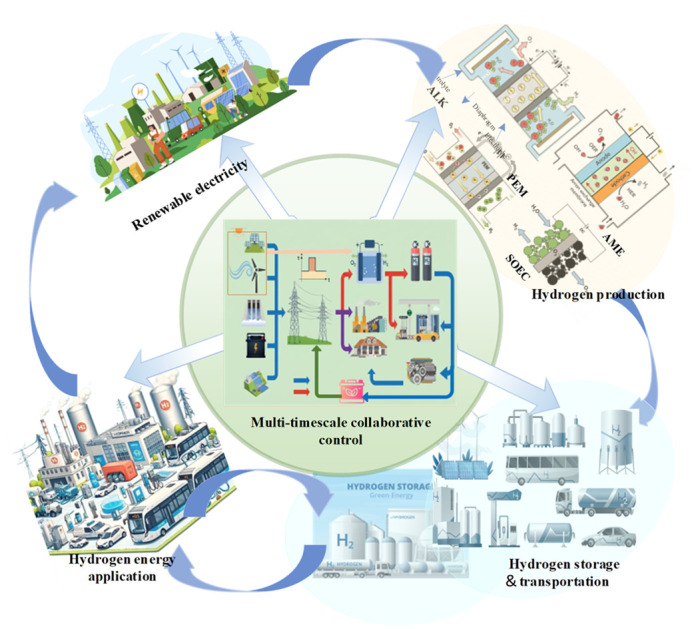

## 1. Introduction

As power systems move toward higher shares of renewable energy, the variability, uncertainty, and seasonal mismatch of wind and solar generation are creating multi-timescale operational challenges, ranging from short-term power fluctuations to intra-day flexibility requirements and inter-seasonal supply-demand imbalances.^1^ The electrochemical storage, particularly battery-based storage, has become an essential flexibility resource for renewable power systems because of its fast response, modular, and effectiveness in frequency regulation, ramp-rate smoothing, peak shaving, and intra-day energy shifting. Conventional peaking resources and short-duration electrochemical storage alone are often insufficient to address these challenges cost-effectively while maintaining capacity adequacy, operational stability, and long-term energy balance. Accordingly, there is a growing need for energy conversion and storage pathways capable of large-scale, long-duration, and cross-regional energy shifting. In this context, electro-hydrogen synergetic systems have emerged as a promising solution because they couple variable renewable electricity with hydrogen production, storage, transport, and end use, thereby enabling both renewable energy integration and multi-sector decarbonization.

Hydrogen is recognized as a secondary energy carrier that can bridge variable renewable electricity and diversified end-use demands, owing to its high gravimetric energy density, multi-form storage options, and long-distance transport capability, as well as its potential for near-zero-carbon utilization in transportation, industry, and buildings.^2,3^ Electro-hydrogen synergistic systems, with water electrolysis as the central hub, convert surplus or low-cost electricity into hydrogen and redistribute energy across time and space through storage, transportation, and reconversion. On the one hand, the system can enhance renewable energy integration, improve grid resilience, and provide ancillary services such as peak shaving and frequency regulation. On the other hand, they offer deep-decarbonization pathways for steelmaking, chemicals, maritime shipping, and heavy-duty transport, thereby enabling a mitigation solution spanning “source–grid–load–storage–hydrogen”.^4,5^

In recent years, key components of electro-hydrogen synergistic systems have advanced significantly. On the hydrogen production side, multiple technology routes—including proton exchange membrane (PEM),^6^ alkaline electrolyzer (ALK),^7^ anion exchange membrane (AEM),^8^ and solid oxide electrolysis cell (SOEC)^9^—have achieved continuous breakthroughs in catalyst and membrane–electrode assembly (MEA) design, system integration, and operational strategies. On the storage and transport side, diverse pathways are being developed in parallel, including high-pressure gaseous storage, cryogenic liquid hydrogen, solid-state storage, liquid organic hydrogen carriers (LOHCs), and underground hydrogen storage.^10,11^ On the utilization side, fuel cells, hydrogen-fired gas turbines, and power-to-X (*e.g.*, green ammonia and green methanol) are promoting hydrogen's transition from an energy storage medium to a cross-sector decarbonization enabler.^12–14^ Concurrently, recent review studies on zinc-based electrochemical energy storage have further elucidated the current state of development in short-duration energy storage materials, encompassing cathode chemistry, stabilization of Zn metal anodes, and electrolyte design strategies. Specifically, ref. 15 provides an advance in cathode materials for rechargeable zinc-ion batteries. Ref. 16 analyzes contemporary and conventional strategies for protecting Zn metal anodes, with a focus on issues such as instability, side reactions, dendritic growth, and cycle degradation. Ref. 17 further synthesizes mechanistic and electrolyte design strategies for zinc metal batteries, emphasizing in particular the role of electrolyte and interfacial engineering in improving Zn deposition/stripping behavior, suppressing side reactions, and enhancing cycling durability. Nevertheless, the trade-off remains unresolved among cost, performance, and lifetime at the materials and component levels; the storage and transport routes face hard constraints among efficiency losses, safety boundaries, and infrastructure compatibility. And the system efficiency and emissions control of downstream conversion pathways must still be coordinated with the upstream characteristics of fluctuating power supply.

More importantly, electro-hydrogen synergistic systems are inherently multi-timescale.^18,19^ At the seconds-to-minutes timescale, it is necessary to mitigate abrupt renewable power ramps and address power-quality and safety risks induced by electrolyzer start–stop behavior and coupling with power electronics. At the hourly to intra-day timescale, economic operation and equipment-friendly scheduling must be achieved under uncertainties in electricity prices, carbon prices, and hydrogen demand. At the weekly to seasonal timescale, inter-seasonal hydrogen storage is required to hedge against structural supply-demand mismatches and to support system capacity adequacy. Therefore, electro-hydrogen synergy is a complex coupled system jointly shaped by materials degradation, dynamic control, market mechanisms, and safety constraints.^20,21^ However, these efforts lack a closed-loop discussion on the coupling relationship among material attenuation, control strategies, and technical economic performance in each stage of electrolysis, storage, transportation, or utilization. The dynamic collaborative optimization and unified evaluation framework for the entire chain performance is still not complete, especially in terms of comprehensive evaluation of life cycle economy, system resilience, and safety, which still needs to be strengthened.

**Fig. 1:**
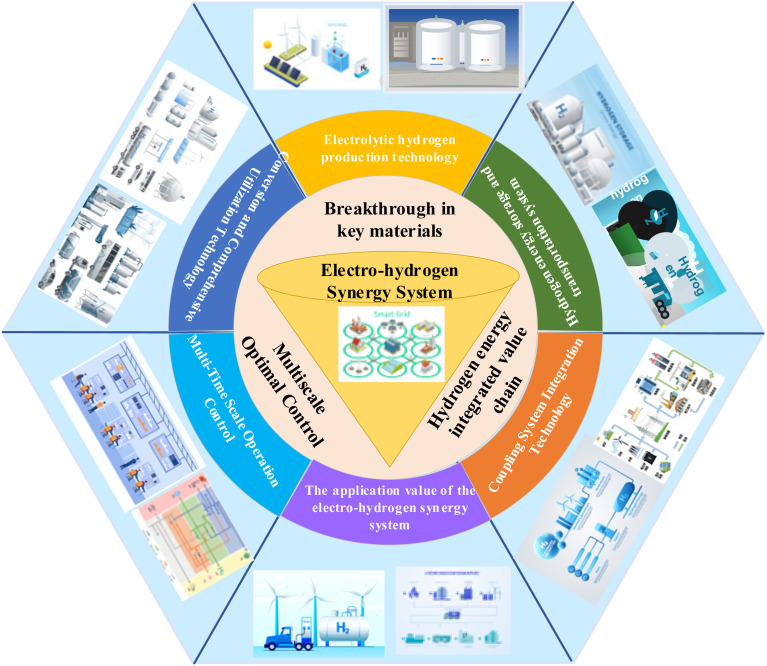
Overall research framework for electro-hydrogen synergistic systems.

Against this backdrop, this review proposes a framework organized around key material breakthroughs, multi-timescale control, and full industrial-chain integration, aiming to connect the critical scientific questions and engineering pathways from microscopic materials to macroscopic energy networks. Fig. 1 presents the overall research framework for electro-hydrogen synergy. First, we synthesize the progress in catalysts, MEAs, and stack-level diagnostics across electrolyzer types, and discuss durability and cost bottlenecks under fluctuating power inputs and industrial current densities. Second, we compare storage and transport technologies, clarifying the applicability boundaries of different routes for long-duration storage and cross-regional supply chains. Third, from the evolution of architectures spanning microgrids, industrial parks, and regional systems, we summarize modeling, control, and dispatch methodologies for coupled multi-energy flows, with an emphasis on multi-timescale optimization strategies for grid ancillary services and seasonal storage, as well as the corresponding validation requirements. Finally, we discuss the application value of electro-hydrogen synergy for renewable energy consumption, grid ancillary services, and decarbonization, outline future research directions for deep-decarbonization applications in transportation and industry, and propose key scientific questions and technical roadmaps to support large-scale demonstration and industrialization. The review seeks to provide a knowledge framework and a research roadmap to support technology development, engineering demonstrations, and large-scale industrial deployment of electro-hydrogen synergistic systems.

## 2. Key technologies for electrolytic hydrogen production: from material breakthroughs to dynamic-response optimization

The electrolyzer is the core unit of green hydrogen production and electro-hydrogen integrated systems. According to the electrolyte and operating conditions, the main technological routes include PEM, ALK, AEM, and SOEC. In recent years, progress has been achieved in catalysts, MEA, and system integration, with advances in low-noble-metal and non-noble-metal electrocatalysts, reinforced or interface-optimized membranes, and *in situ* diagnostic and control strategies improving electrolyzer activity, stability, and operational adaptability under practical conditions.^22–42^ Nevertheless, these technologies still face a trade-off among cost, efficiency, and durability, as PEM relies on Ir- and Pt-based catalysts, ALK generally suffers from relatively slow dynamic response, AEM is constrained by membrane stability and interfacial compatibility, and SOEC remains challenged by material degradation under high-temperature operation. Especially under the fluctuating power supply of wind and solar energy, electrolyzers need to balance high efficiency and rapid dynamic response, promoting the transformation of material performance to reliable operation at system levels. In addition, under fluctuating wind and solar power input, electrolyzers must simultaneously maintain high conversion efficiency and rapid load-following capability while mitigating degradation induced by start–stop operation, variable load, and thermal/electrochemical stresses. Therefore, further efforts are required to promote the translation of material-level advances into reliable stack- and system-level operation through coordinated development of materials, MEAs, diagnostics, and dynamic control strategies. To provide an overview of technological routes for electrolytic hydrogen production, Table 1 compares PEM, ALK, AEM, and SOEC in terms of representative material advances, dynamic-response characteristics, major advantages, and current scale-up bottlenecks.

**Table 1:** Comparative summary of key electrolytic hydrogen production technologies

Technology	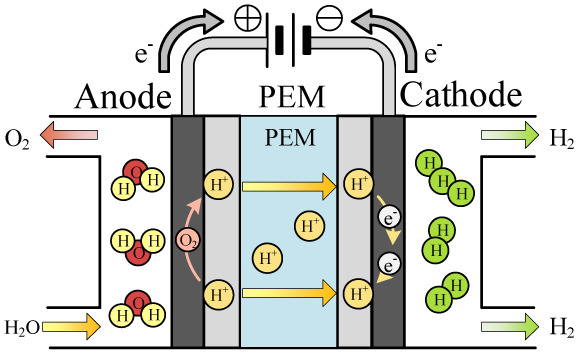	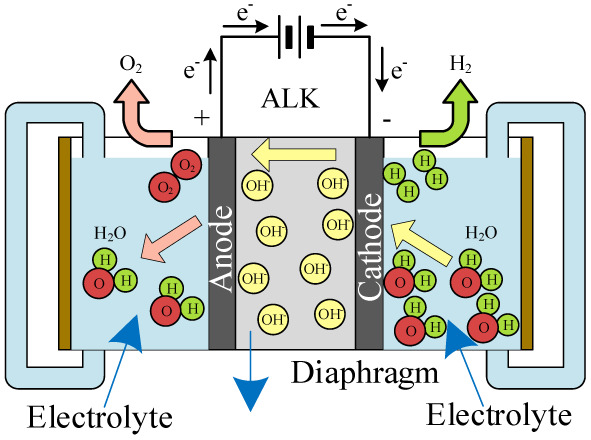	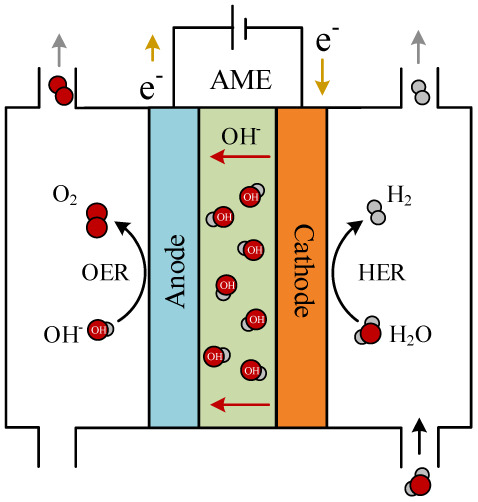	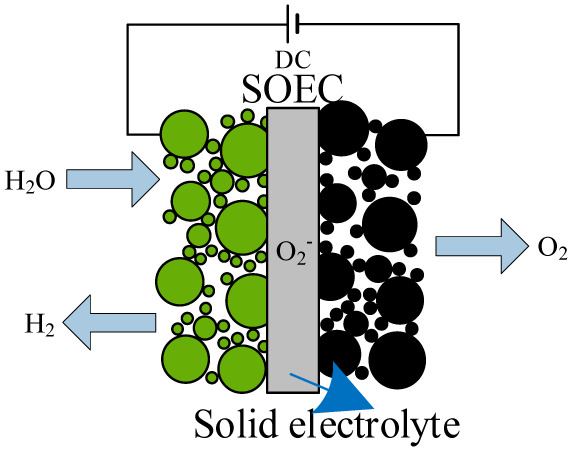
Typical operating conditions	Acidic solid polymer electrolyte; typically low-temperature operation	Liquid alkaline electrolyte; mature low-temperature route	Alkaline membrane environment; low-temperature operation combining membrane architecture with non-precious-metal catalysis	High-temperature solid oxide electrolysis
Representative material/system advances	Low-Ir amorphous IrO_2_ nanoclusters for enhanced acidic OER; sub-nanometric Pt clusters on hierarchical porous supports for improved HER; *in situ* stack-level diagnostic methods for MEA inconsistency evaluation	Bifunctional transition-metal catalysts, electrode/interface engineering, and self-supported catalytic layers to reduce overpotential under industrial current density	Reinforced anion exchange membranes with improved conductivity and dimensional stability; catalyst optimization based on spinel and mixed-metal oxides	Metal-supported SOCs, low-Ni fuel electrodes, and emerging CO_2_/co-electrolysis catalyst systems; exploration of direct electrolysis for complex water sources
Dynamic-response characteristics	Fast dynamic response, well-suited for intermittent wind/solar coupling and rapid load-following operation	Moderate/slower dynamic response than PEM; dynamic adaptability still needs improvement under highly fluctuating power input	Potentially faster than ALK and lower-cost than PEM, but current stability remains limited	Thermally constrained response, less flexible than low-temperature routes, but attractive under stable high-temperature integration scenarios
Major advantages	High current density, compact design, high-purity hydrogen output, strong compatibility with fluctuating renewable electricity	Technologically mature, relatively low cost, and easier large-scale deployment	Potential use of non-precious-metal catalysts, promising a balance between cost and performance	High theoretical efficiency, strong potential for heat integration, syngas/co-electrolysis capability
Main bottlenecks for scale-up	Dependence on scarce Ir/Pt catalysts, high cost, MEA inconsistency within stacks, insufficient long-term validation under start–stop/variable-load conditions	Sluggish dynamic response, gas crossover risk at partial load, catalyst synthesis complexity, insufficient understanding of degradation under fluctuating conditions	Membrane conductivity and alkaline stability, interfacial compatibility, insufficient durability under realistic stack operation, limited large-area MEA validation	High-temperature material degradation, thermal cycling instability, insufficient large-scale stack validation, limited evidence for long-term engineering durability
Representative references	22–29	30–34	35–38	39–42

### 2.1. Innovation and performance improvement of key materials for PEM

PEM is regarded as a promising technology for green hydrogen production because it combines compact system design, high operating current density, rapid load-following capability, and high-purity hydrogen output, making it particularly suitable for coupling with intermittent renewable electricity.^22,23^ However, its large-scale deployment is still constrained by both material-level bottlenecks. Under acidic operating conditions, the anodic OER still relies predominantly on Ir/IrO_*x*_-based catalysts because few non-noble materials can simultaneously satisfy activity and corrosion-resistance requirements, whereas the cathodic HER generally depends on Pt-based catalysts, resulting in persistent concerns regarding noble-metal cost, resource scarcity, and long-term durability.^24,25^ In addition, nonuniformities in MEA fabrication, compression, interfacial contact, and local reactant distribution can induce cell-to-cell performance inconsistency within PEM stacks, amplify local ohmic and mass-transport losses, and hinder scale-up and stable industrial operation.^26^ The studies have made progress toward these challenges. Ref. 27 synthesized low-loading amorphous Ir nanoclusters through quasi-room-temperature high-pressure H_2_ reduction and showed that the surface amorphous IrO_*x*_ structure enhanced acidic OER activity. Ref. 28 developed a hierarchical micro/nano-porous Co aerogel supporting sub-nanometric Pt clusters, which improved three-phase mass transfer and Pt–Co interaction and sustained more than 200 h of HER operation at industrial current density in a PEM electrolyser. Ref. 29 further proposed a micro-current-excitation-based *in situ* diagnostic method capable of extracting hydrogen crossover current, double-layer capacitance, and ohmic resistance for individual MEAs. Nevertheless, current evidence remains insufficient for long-term validation under realistic start–stop, variable-load, and other industrial boundary conditions, while the links among accelerated stress testing, large-area MEA consistency, and diagnostic feedback are still underdeveloped.

### 2.2. Dynamic response enhancement and material optimization of ALK

ALK is mature and has low cost, but its dynamic response is relatively sluggish. In terms of bifunctional catalysis, ref. 30 constructed Se-modulated and V-doped CoMoO_4_ nanospheres, which delivered overpotentials of 213 mV for OER and 142 mV for HER at 10 mA cm^−2^ and sustained 50 mA cm^−2^ for 20 h with negligible potential decay. Ref. 31 demonstrated self-supported V-doped Cu_2_S with a geometric current density of more than 500 mA cm^−2^ current for bi-functional electrolysis in an industrial-scale alkaline electrolyte 5 M KOH at an elevated temperature of 60 °C. Regarding the engineering modification of HER, ref. 32 constructed a Co–Co(OH)_2_ catalytic layer on foam nickel, the electrocatalysts lowered the cell potential by 200 mV at 200 mA cm^−2^ compared to pristine nickel foam after 118 h of electrochemical measurements at 80 °C using a 30 wt% KOH electrolyte solution. Simultaneously, ref. 33 expanded the HER design concept by modifying Pd on MoS_2_/CoS_2_ and strengthening the metal–support interaction. While for OER, ref. 34 constructed FeNi nanoparticles/reduced graphene oxide on FeNi LDH supported by Ni foam, achieving an overpotential of 234 mV at 10 mA cm^−2^, which was 37 mV lower than that of commercial RuO_2_ on Ni foam, while maintaining stable operation for over 1300 h and withstanding 10 000 accelerated degradation cycles. These references were verified under high temperature, high alkalinity, and high current density conditions, and presented long-term stable samples. However, the complexity of synthesis and consistency of large-scale production of most high-performance catalysts remain challenges, and the attenuation mechanism and dynamic response improvement paths under fluctuating conditions and full cell integration conditions are still unclear. Moreover, the understanding of active site identification and the synergy mechanism of composite catalysts can be further deepened.

### 2.3. Research on material bottlenecks and stability of AEM

The AEM technology has attracted attention due to its ability to use non-precious metal catalysts. However, its development is still limited by challenges such as the ion conductivity, dimensional stability, and the synergistic integration with efficient non-precious metal catalysts of the anion exchange membrane. Recent studies have made progress in both membrane material design and catalyst optimization.^35,36^ In membrane-oriented studies, researchers have focused on introducing quaternary ammonium functional groups, reinforcement frameworks, and interfacial regulation strategies to improve anion conductivity, suppress membrane swelling, and enhance dimensional and mechanical stability. Literature^35^ introduced quaternary ammonium groups such as DABCO, TMA, and methylated DABCO into poly(*meta*-terphenylene)-based anion exchange membranes and reinforced them with PPS fiber mats to suppress swelling. Among these, the PPS/mTPN/DABCO-Me membrane achieved the highest OH^−^ conductivity, reaching 140 mS cm^−1^ in 1 M KOH and 237 mS cm^−1^ in 4 M KOH, while an electrolyzer using this membrane maintained a stable cell voltage of 1.9 V at 0.25 A cm^−2^ for 100 h at 60 °C in 4 M KOH without detectable membrane resistance change. To further address the interface issues caused by the difference in swelling behavior between the reinforcing agent and the matrix, literature^36^ covalently connected polybenzimidazole nanofiber mats with reactive amine groups to the matrix to alleviate interface mismatch and improve mechanical/dimensional stability, achieving stable operation for approximately 200 hours. However, the long-term alkaline stability of membrane materials, especially the chemical decay behavior of quaternary ammonium groups in a high-temperature and strong-alkali environment, has not been fully verified. In catalyst-oriented studies, efforts have mainly centered on the development of spinel-based and mixed-metal oxide electrocatalysts through compositional tuning, doping, and surface engineering to improve oxygen evolution activity and to better understand active-site mechanisms.^37,38^ Ref. 37 indicates that spinel-type materials are considered promising oxygen evolution reaction catalysts due to their inherent catalytic properties and abundance, and their performance can be further optimized through doping and surface engineering. Literature^38^ further demonstrated that there is an optimal Co/Cu ratio for copper cobalt mixed oxides to maximize battery performance and explore the mechanism of active sites. However, most of these catalysts remain at the material/semi-battery level and require long-term verification in large-area MEA preparation, compatibility with membranes/ion conductors, and real AEM stack/slot operating conditions.

### 2.4. SOEC and emerging electrolysis technologies: high temperature and diversified applications

The multiple pathways of solid oxide electrolysis, carbon dioxide reduction, and direct electrolysis of complex water sources provide supplements to green energy. Recent research has made a series of progress in expanding the performance, stability, and applicability of these systems. Ref. 39 constructed a metal-supported SOC fuel electrode using low Ni and permeable GDC, achieving initial performance and durability comparable to or even better than commercial ceramic batteries in co-electrolysis, providing a robust cathode solution for green synthesis gas production. To reduce precious-metal usage while improving catalytic performance, ref. 40 employed 4-aminobenzothiophenol as an anchoring agent to prepare evenly dispersed Ag nanoparticles with an average particle size of 2 nm, achieving a current density of 1 A cm^−2^ with faradaic efficiency for CO of 85% at 3.3 V. These results were obtained with a low Ag loading of 0.26 mg_Ag_ cm^−2^, yielding a turnover frequency of 9.6 s^−1^. In addition, the catalyst maintained CO faradaic efficiencies of around 80% for exceeding 200 h at 500 mA cm^−2^, with a cell-voltage degradation rate of 1.5 mV h^−1^. However, these frontier explorations still have the problem of insufficient long-term stability verification time for metal-supported batteries and CO_2_ electrolysis catalysts, and the test scale is limited to laboratory-scale single cells or electrodes. For non-ideal water sources, ref. 41 proposed a strategy of integrating forward osmosis and phosphate buffer electrolysis, effectively extracting pure water from seawater and *in situ* electrolyzing hydrogen production, and using non-precious metal porous nickel-based catalysts to achieve continuous 6-day stable operation in a custom electrolyzer. To address multifunctional electrocatalysis in complex aqueous matrices, ref. 42 achieved a self-supported P–SnCoFe-LDH/NF architecture engineered through dual-ion co-doping, demonstrating unprecedented performance in chloride-rich alkaline oilfield wastewater. The dual electronic synergy establishes optimal adsorption–desorption equilibrium, evidenced by 21% reduced OER charge transfer resistance and 56% enhanced HER Volmer kinetics.

## 3. Hydrogen storage and transportation system: material innovation and multi-scale synergistic optimization

### 3.1. Optimization of storage materials and system performance: from high-pressure gaseous to solid-state storage

**Fig. 2:**
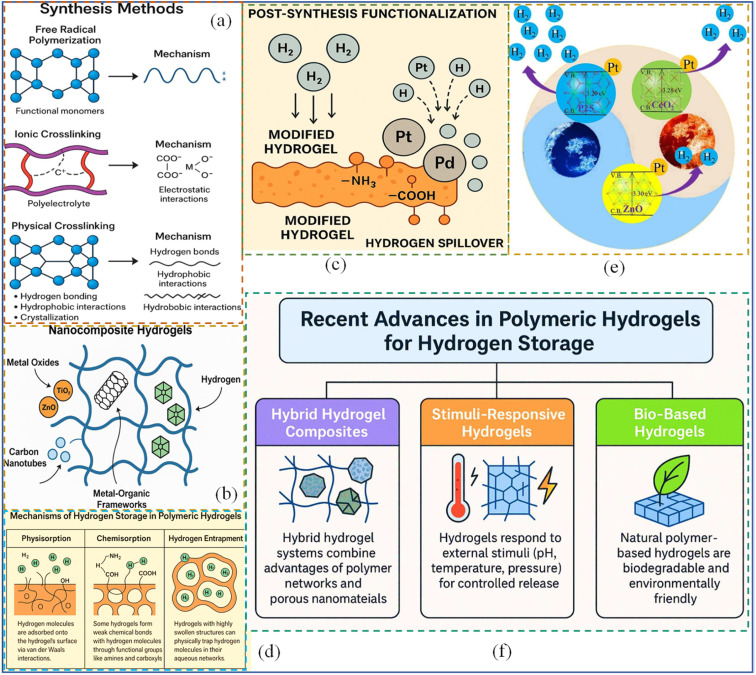
(a) Overview of water gel synthesis methods and interaction mechanisms. (b) Schematic diagram of a nanocomposite water gel. (c) Synthesis of water gel. (d) Hydrogen storage mechanism in polymer water gel. (e) Cooperative hydrogen evolution reaction using platinum-modified semiconductor photocatalyst. (f) Latest strategies for hydrogen storage using polymer water gel. Adapted from ref. 50 with permission from Elsevier: Chandra Sekhar Espenti, *et al.*, *International Journal of Hydrogen Energy*, 2025, **158**, 150 529. Copyright © 2025 Hydrogen Energy Publications LLC.

Hydrogen energy storage technology is shifting from a single solution competition to diversified development, scenario-based application, and system integration. High-pressure gaseous hydrogen storage has high maturity and is suitable for offshore/demonstration projects, but it has low volumetric energy density and suffers from leakage and high-pressure safety risks, which limit its promotion on large-scale ocean-going vessels.^43,44^ Low-temperature liquid hydrogen storage has density advantages and is an important form of cross-regional trade, but it has high liquefaction energy consumption and continuous evaporation losses.^45^ Solid-state hydrogen storage has high volumetric density and inherent safety, and nano-structure regulation and catalytic doping can improve capacity and kinetics.^46,47^ However, the stress accumulation caused by metal hydride cycling expansion may lead to reactor damage,^48^ and porous materials have shortcomings in room-temperature weight capacity and cycling stability.^49^ Additionally, flexible hydrogen storage materials such as polymer hydrogels have limited capacity but outstanding processability and safety, providing a new path for flexible devices,^50^ as shown in Fig. 2. Liquid organic hydrogen carriers can utilize existing oil infrastructure for long-distance, large-scale, safe transportation, but dehydrogenation energy consumption and catalyst costs remain economic bottlenecks.^51,52^ Underground hydrogen storage has great potential for long-term large-scale energy storage, but its efficiency and safety depend on the gas–water–rock coupling mechanism and reservoir integrity, and long-term stability needs to be verified.^53^ But the existing research lacks a system comparison of the synergistic and complementary potential among different technologies, full life-cycle economic assessment, and dynamic matching mechanisms in large-scale integration applications. Systematic research across technologies and multiple scales is needed to promote the overall optimization and practical application of hydrogen energy storage technology.

From the perspective of system integration and optimization, the research focus is shifting from improving component performance to collaborative design and operation optimization for production, storage, transportation, and application. The framework for the collaborative optimization of the liquid organic hydrogen carrier storage and transportation system and the renewable energy-rich power grid indicates that coordinated operation can significantly enhance the economic and reliability of the system.^54,55^ Multi-scale numerical simulations are becoming key tools, and machine learning can accelerate material screening and be used for intelligent monitoring and predictive maintenance of hydrogen storage equipment. Meanwhile, multi-scale numerical simulation techniques such as computational fluid dynamics, finite element analysis, and molecular dynamics can connect molecular adsorption, heat and mass transfer, and structural response, providing a basis for tank and reactor design and process optimization.^56^ To make trade-offs in technical, economic, environmental, and even social-political factors, multi-criteria decision-making methods have been widely applied to select the optimal technical path.^57^

Considering the application scenarios and economic viability of integrated coupling systems, the maritime industry is a typical application field for hydrogen storage and also a place where its storage technologies can be tested. Fuel cells require a long service life, and the hydrogen storage system must also meet the space limitations of ships and higher safety requirements.^58^ Literature^59,60^ indicates that the cost of hydrogen storage varies, with capital expenditure, scale, and transportation distance being the main factors. Literature^61,62^ further indicates through life cycle assessment that the hydrogen emission reduction benefits depend on the upstream hydrogen supply structure. Therefore, based on the existing local combination or static scenario studies, an attempt can be made to construct a dynamic coupling and collaborative optimization model covering the production-storage-transportation-application chain. By using data-driven and simulation techniques and other methods, the reliability, generalization ability, and deep integration with actual engineering data of the system model can be strengthened.

### 3.2. Hydrogen transportation technology: multi-mode synergy and economic analysis

**Fig. 3:**
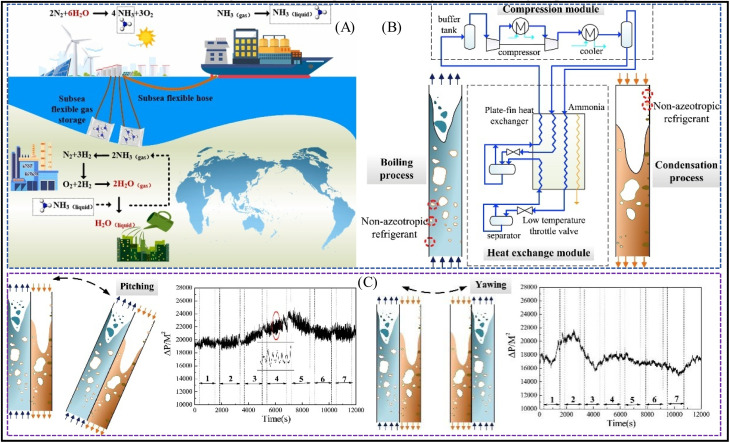
(A) New marine ammonia–hydrogen coupling storage and transportation technology; (B) low-temperature heat transfer process in the liquid ammonia temperature zone; (C) pressure drop performance under different scenarios. Adapted from ref. 74 with permission from Elsevier: Chongzheng Sun, Xin Fan, Yuxing Li, *et al.*, *Renewable Energy*, 2022, **201**, 700–711. Copyright © 2022 Elsevier Ltd.

The large-scale utilization of hydrogen requires the support of an efficient and safe multi-mode transportation system. Compressed gas hydrogen has the advantage of simple infrastructure and low cost, and thus has economic benefits in short-distance transportation,^63,64^ but its low energy density and high pressure risks limit its large-scale application.^65,66^ Liquid hydrogen increases density through liquefaction and is suitable for long-distance transportation, but the liquefaction process has high energy consumption, accounting for 30–40% of the production cost, and there is severe evaporation loss during storage.^67^ Cryogenic compressed hydrogen strikes a balance between density and cost, but is still insufficiently mature. Solid hydrogen storage has high safety but still faces challenges in kinetics and material costs.^68,69^ Organic liquid hydrogen has the advantages of high safety in storage and transportation, long-distance transportation capability, and high hydrogen storage density. However, hydrogen dehydrogenation requires energy consumption and catalysts, the system efficiency will decrease, and the device is more complex.^70,71^ The economic performance of transportation methods is strongly coupled with distance, scale, and discount rate. Pipeline costs increase with distance, while ship transportation is suitable for international long-distance transportation but has a high initial investment.^72,73^ Literature^74^ demonstrates the potential for synergy between electricity–hydrogen/ammonia–hydrogen, such as the offshore ammonia–hydrogen coupling storage through the LNH_3_ floating production, storage, and offloading unit to enhance adaptability to complex oscillating conditions, but energy consumption and costs still need to be optimized, as shown in Fig. 3. The transportation hub superconducting hydrogen-electric multi-energy system can improve transmission efficiency and reduce losses, but it is also constrained by capital investment.^75^ In summary, the cost of hydrogen storage and transportation is influenced by multiple factors such as capital expenditure, scale, distance, and discount rate. Currently, the cost of green hydrogen storage and transportation is still high, but there is a potential for reduction in the future. Research on supply chain design indicates that introducing liquid organic carriers and other alternative options can reduce carbon emissions, but they are influenced by policy regulations such as carbon taxes.^76^ This highlights that policy orientation and public acceptance are also key variables. Carbon taxes can effectively drive the selection of low-carbon technologies, and the enhancement of public awareness can help increase willingness to pay. However, existing research focuses more on specific technical paths or static scenarios and lacks comprehensive, multi-dimensional dynamic comparison and integration evaluation of different storage and transportation technologies, especially the reliability of the large-scale application of emerging technologies, the real-time coordination with intermittent renewable energy, and the supply chain resilience under policy changes.

**Fig. 4:**
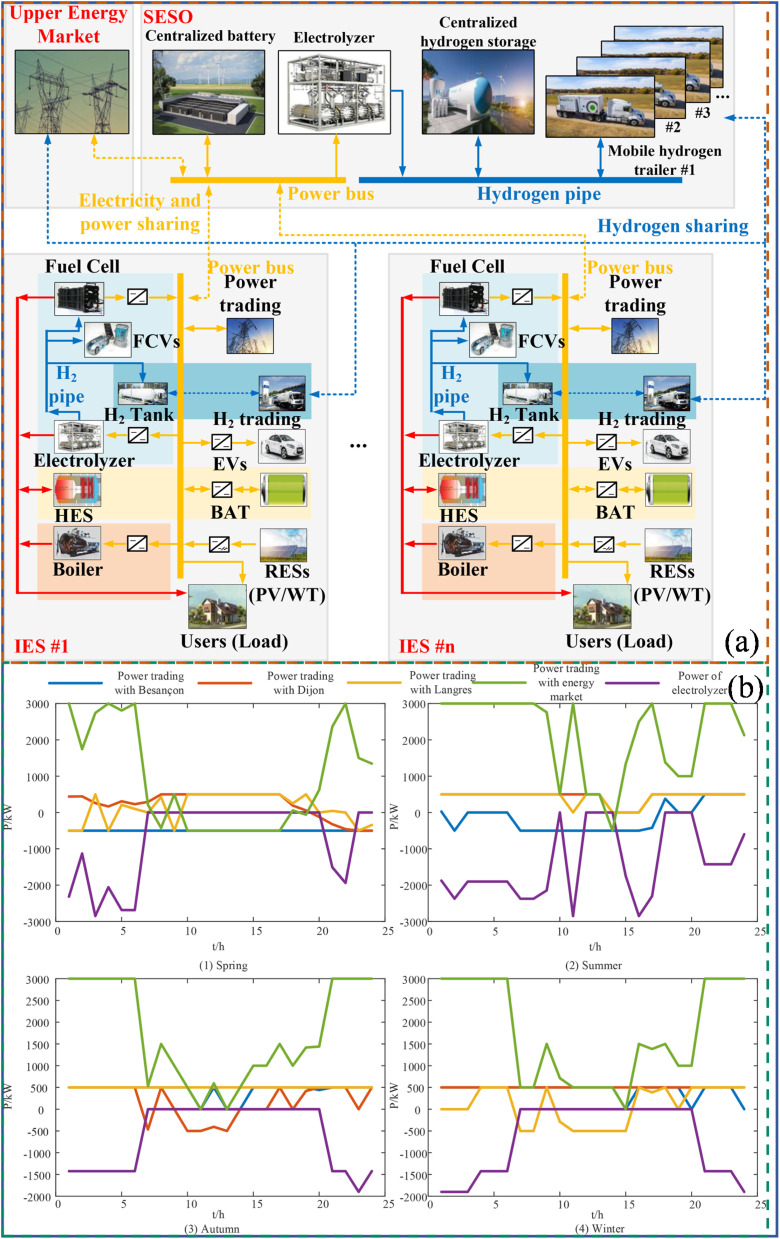
(a) Topology of the regional energy system; (b) power trading of IESs. Adapted from ref. 81 with permission from Elsevier: Yuchen Pu, Qi Li, Shasha Huo, *et al.*, *Renewable Energy*, 2024, **237**, Part C, 121 828. Copyright © 2024 Elsevier Ltd.

The current common challenges in hydrogen energy storage and transportation technology mainly lie at the material and system ends, including hydrogen permeation through polymer barriers, the cyclic stability of storage materials, the energy conversion efficiency of the link, and the risks of hydrogen embrittlement and leakage.^77^ Material compatibility assessment shows that PEEK and polyimide perform better in high-pressure hydrogen environments, while PTFE is more suitable for sealing systems, providing a basis for engineering material selection.^78^ Further, at the system level, the overall efficiency and economy of the renewable energy – green hydrogen – renewable energy chain still lack appeal, and the safety assessment tools and unified standard system still need to be improved. At the same time, hydrogen as an alternative fuel is still constrained in its production, storage, and transportation chain by high green hydrogen costs, low hydrogen density, and insufficient infrastructure. From an economic perspective, the intensity and scale of infrastructure investment are uneconomical, resulting in high storage costs, especially in developing countries. The cost sensitivity analysis confirms that capital expenditure, transportation distance, and discount rate are the core levers, but some studies have also found that in grid-constrained scenarios, hydrogen fuel cell vehicles can form load complementarity with pure electric vehicles and can promote traffic emission reduction without capacity expansion.^79^ From a policy perspective, the lack of global unified technical standards and the fragmentation of business models have hindered the integration of the industrial chain 80. Ref. 81 points out that enhancing barrier performance through polymer composites and nanofillers can be a focus in the future, and analyzing their failure mechanisms. Moreover, a collaborative architecture for electric hydrogen shared storage and mobile transportation is constructed to improve resource utilization, as shown in Fig. 4. The strengthening of scenario-oriented routes of decentralized hydrogen production and ammonia–hydrogen coupling, and promoting large-scale implementation through carbon pricing and standardization.^82^ However, although existing research has made progress in many aspects, it still lacks in-depth exploration of the dynamic interaction effects of complex systems. There is insufficient research on differentiated paths in different regional scenarios, especially in developing countries, and it is urgently needed to establish a research framework for the entire chain, full life cycle, and dynamic uncertainty of the electric-hydrogen collaborative system, integrating material innovation, system optimization, and engineering verification to support safe, economic, and low-carbon operation under a high proportion of renewable energy scenarios.

Beyond techno-economic performance, the industrial deployment of electro-hydrogen systems is strongly constrained by material compatibility, operational safety, and regulatory readiness.^83^ In particular, when hydrogen is transported through repurposed natural gas infrastructure, the compatibility of transmission steels, polymer liners, elastomeric seals, compressors, and end-use equipment must be systematically evaluated under hydrogen-containing environments, because hydrogen may alter fracture behavior, accelerate permeability-related degradation, and affect combustion stability in downstream applications.^84^ Hydrogen embrittlement remains a critical concern for pipeline steels and high-pressure metallic components, and current assessment approaches still lack standardized, quantitative, and harmonized testing procedures for material susceptibility and fitness-for-service.^85^ In parallel, hydrogen leakage cannot be treated merely as a component-level sealing issue, it should be incorporated into integrated safety protocols covering leak detection, ventilation design, ignition prevention, emergency shutdown, consequence analysis, and quantitative risk assessment across storage, transport, refueling, and end-use scenarios.^86,87^ Moreover, it is necessary to conduct further in-depth research on aspects such as hydrogen gas mixing limits, qualification procedures for aged natural gas assets, harmonization of cross-regional testing methods, and coordination of code, standards, and licensing requirements in industrial hydrogen projects. From a multi-dimensional perspective of integrating cracking and leakage, a comprehensive framework should be constructed that interconnects material certification, infrastructure integrity assessment, safety management, and regulatory standardization, in order to provide a more practical foundation for the large-scale deployment of electro-hydrogen systems.

## 4. Hydrogen conversion and comprehensive utilization technology: from hydrogen to electricity and multi-site coupling

### 4.1. Fuel cell technology: material durability, system integration, and transportation applications

**Fig. 5:**
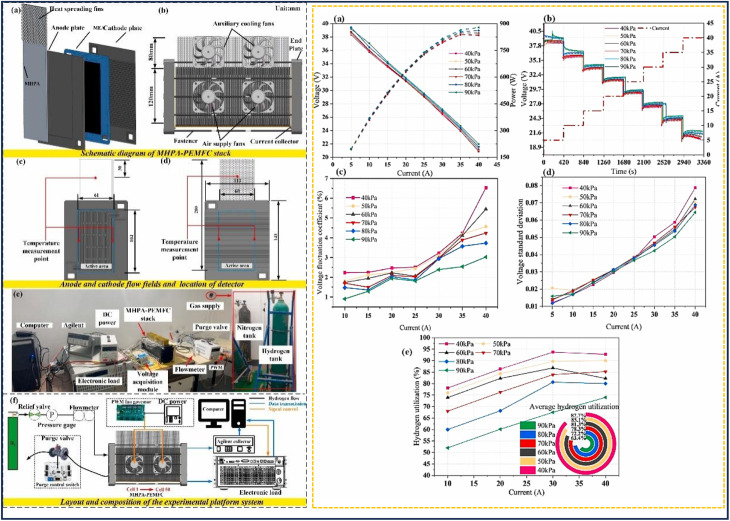
Composition of MHPA-PEMFC stack and the experimental platform system. (a) MHPA bipolar plate, (b) MHPA-PEMFC stack, (c) anode flow fields and the location of the detector, (d) cathode, (e) overview of the experimental platform, (f) schematic diagram of the experimental platform. Characteristics of the stack under different hydrogen supply pressures. (a) Polarization curve, (b) instantaneous voltage, (c) voltage fluctuation coefficient, (d) standard deviation of cell voltage, (e) hydrogen utilization rate. Adapted from ref. 90 with permission from Elsevier: Zejian Chang, Zhenhua Quan, Mingguang Yang, *et al.*, *Journal of Power Sources*, 2026, **661**, 238 636. Copyright © 2025 Elsevier B.V.

The fuel cells are the core component of the hydrogen-to-electricity conversion unit in the electro-hydrogen integrated system, and they significantly affect the efficiency, cost, and reliability of the entire industry of renewable energy hydrogen production, storage, and utilization. Currently, proton exchange membrane fuel cells (PEMFC) and solid oxide fuel cells (SOFC) constitute two main routes: PEMFC is more suitable for dynamic conditions such as transportation, while SOFC is more applicable to fixed power generation and multi-fuel coupling scenarios.^88,89^ PEMFC has the advantages of rapid startup and high power density, but its performance is highly sensitive to operating strategies and material durability. For air-cooled PEMFC stacks, optimizing hydrogen supply pressure and purging strategies can significantly suppress voltage fluctuations.^90^Fig. 5 shows the composition of the MHPA-PEMFC module and the experimental platform system: (a) MHPA bipolar plates, (b) MHPA-PEMFC module, (c) anode flow field and detector position, (d) cathode, (e) overview of the experimental platform, (f) schematic diagram of the experimental platform; characteristics of the module under different hydrogen supply pressures: (a) polarization curve, (b) instantaneous voltage, (c) voltage fluctuation coefficient, (d) standard deviation of battery voltage, (e) hydrogen utilization rate. Meanwhile, hydrogen–oxygen fuel cells have a higher degradation risk in pure oxygen environments, and water management and key material durability become key constraints for improving reliability.^91^ PEMFC has the advantages of rapid startup and high power density, but it relies on precious metal catalysts and has insufficient durability. Furthermore, SOFC, with its high efficiency and fuel flexibility, can further enhance system energy efficiency and economic performance in hydrogen/ammonia/methanol carrier coupling and waste heat recovery integration.^92^ The integrated system of the high-temperature cracker and solid oxide fuel cell achieves the highest efficiency of 69.55% and the lowest levelized cost of electricity at 0.145 USD per kWh, ammonia's potential as a cost-effective hydrogen carrier, particularly in renewable-rich regions for large-scale ammonia synthesis and export to high energy cost markets.^93^

**Fig. 6:**
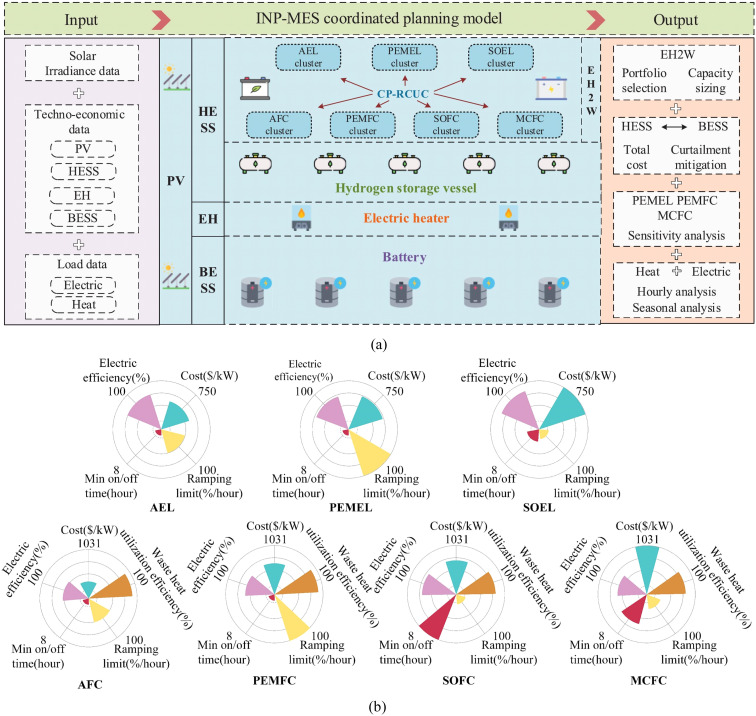
(a) The overall framework of the study(industrial park multi-energy systems). (b) Comparison of techno-economic metrics for different hydrogen-to-water conversion technologies. Adapted from ref. 94 with permission from Elsevier: Jiahui Liang, Shaocong Wu, Tianguang Lu, *Applied Energy*, 2026, **403**, Part B, 127 001. Copyright © 2025 Elsevier Ltd.

For high penetration of renewable energy, the coordinated planning and scheduling of fuel cells, electrolysis for hydrogen production, hydrogen storage, and thermal utilization can effectively balance intermittency and enhance power supply reliability. The coordinated planning of multi-energy systems in industrial parks indicates that by optimizing the combination of heterogeneous electrolyzers and fuel cells and configuring a hydrogen energy storage system, the rate of power loss can be reduced and system costs can be decreased,^94^ as shown in Fig. 6. However, compared to lithium-ion batteries, the hydrogen path has advantages of long-term energy storage and high energy density, but the conversion efficiency from electricity- hydrogen-electricity is low. There are many real-time scheduling variables and complex safety constraints, increasing the difficulty and uncertainty of operation optimization.^95^ Hydrogen fuel cell systems have shown promising application prospects in both air and ground transportation fields, and they have obvious advantages in terms of range, convenient refueling, and low carbon emissions.^96,97^

The application of hydrogen fuel cell technology in the transportation is expanding from road vehicles to various other fields, such as aviation and maritime. Ref. 98 states that when hydrogen fuel cell truck costs drop from 35 CNY per kg to 20 CNY per kg, the number of trucks expected to be on the road by 2035 goes from less than 0.6 million to more than 1.2 million. Lifecycle analysis shows that while implementing a 20% purchase subsidy, HFCV's LCC will become more competitive than EVs by 2030.^99^ In the aviation and maritime scenarios, the volume/weight of hydrogen storage and safety become the constraints, and the compatibility assessment of onboard hydrogen storage and fuel cell propulsion systems generally emphasizes storage density, layout, and certification difficulty.^100,101^ The dual-mode integration of fuel cells and gas turbines can achieve fuel consumption reduction, but the power coupling and control are complex.^102^ The hydrogen-electric hybrid system for unmanned aerial vehicles can extend the range, but the real-time adaptability of energy management still needs to be improved.^103^ In terms of safety, the diffusion and explosion risks of hydrogen leakage in enclosed or semi-enclosed spaces are the key barriers for large-scale deployment, and existing research mostly relies on CFD and experiments to guide ventilation and sensor layout, but real-time monitoring, dynamic warning, and emergency linkage in complex conditions are insufficient.^104–107^ Moreover, the supply risk of key materials and the fragmentation of standard systems may form an industrial bottleneck, it is urgent to promote alternative materials, recycling, and standard coordination.^108,109^ Some studies start from different scenarios, they all show that the existing risk prevention strategies have deficiencies in real-time, adaptability, and system synergy.^110,111^ Especially, the research on dynamic monitoring and emergency response mechanisms under multi-factor coupling conditions is insufficient, which restricts the large-scale safe deployment of hydrogen fuel cell vehicles in complex environments.^112–114^

Based on the above studies, fuel cell technology is mainly concentrated in specific application fields, which leads to a lack of systematic comparison among different technical paths in terms of lifecycle cost, dynamic response, and multi-scenario adaptability. The comprehensive research pays insufficient attention to the real-time coordinated scheduling of the power-hydro-heat multi-energy flow and its related safety boundaries under the integration of high penetration renewable energy. Moreover, the research on the material degradation mechanism under complex operating conditions needs to be further explored in depth.

### 4.2. Hydrogen gas turbine technology: combustion optimization and multi-energy coupling systems

In electro-hydrogen synergistic systems, hydrogen gas turbines represent a key power-regeneration technology that links hydrogen production, storage, and electricity supply. Surplus renewable electricity can be converted into hydrogen through electrolysis, stored over medium- to long-term timescales, and then reconverted into electricity by hydrogen-based gas turbines during peak-load periods or renewable power shortages. Therefore, hydrogen gas turbines are not only end-use devices for hydrogen combustion, but also important components of power-to-hydrogen-to-power chains and multi-energy coupling systems. At the combustion level, hydrogen micronix combustion reduces NO_*x*_ by shortening the mixing scale and decreasing the combustion zone length, though flashback risks and injector configuration optimization remain critical.^115^ Mixing hydrogen with natural gas can provide a practical compromise between the feasibility of fuel supply and the performance of gas turbines. Natural gas ensures the reliability of fuel supply and is compatible with the existing gas turbine infrastructure; while hydrogen can enhance combustion reactivity, flame propagation speed, output power and efficiency, and reduce the carbon intensity of the fuel mixture.^116,117^ Specifically, hydrogen–natural gas blending enables partial decarbonization while retaining the advantages of existing natural gas infrastructure and fuel availability, and hydrogen addition can also improve flame reactivity. By contrast, hydrogen–ammonia blending takes advantage of the ease of ammonia storage and transport, while hydrogen compensates for the low flame speed and poor ignition characteristics of ammonia, thereby improving flame stability. However, high hydrogen ratios may elevate NO_*x*_ emissions and exacerbate corrosion, necessitating coordinated control strategies such as staged combustion, selective catalytic reduction (SCR), or plasma-assisted ignition.^118–120^ Furthermore, through numerical simulation, the combustion characteristics of hydrogen, ammonia, and hydrogen–ammonia mixed fuels in micro gas turbines were investigated. The hydrogen–ammonia mixture achieved a balance between performance and emission reduction, demonstrating its reliability in cleaner gas turbine technologies. However, the development of ammonia fuel gas turbine technology still faces many challenges, such as increased nitrogen oxide emissions, poor combustion performance, insufficient ignition stability, and material performance degradation. Technical obstacles such as nitrogen oxide control and combustion stability still need to be overcome.^121,122^

**Fig. 7:**
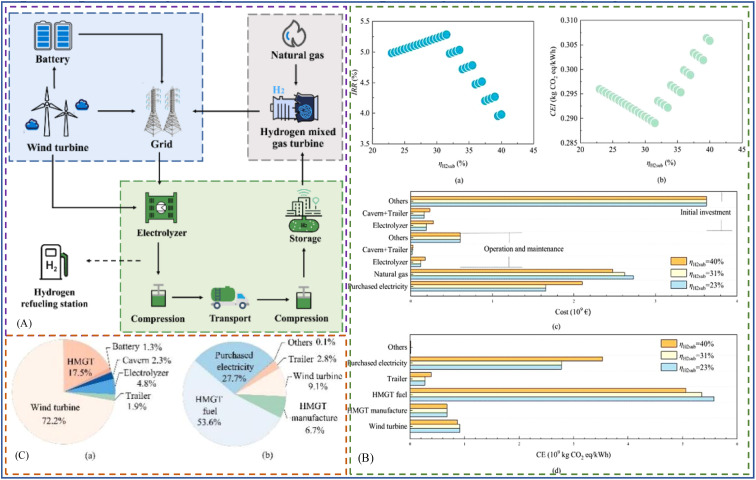
(A) PtH2tP system configuration; (B) influences of different *η*_H2sub_ values on the (a) IRR, (b) CEI, (c) cost and (d) carbon footprint; (C) impact of different devices of the PtH2tP system on the (a) initial investment, and (b) CEs. Adapted from ref. 124 with permission from Elsevier: Yanxin Li, Xiaoqu Han, Yiming Zhang, *et al.*, *International Journal of Hydrogen Energy*, 2025, **145**, 345–357. Copyright © 2025 Hydrogen Energy Publications LLC.

At the system level, the coupling of renewable electricity and hydrogen-based gas turbines has become an important path for building a power-to-power energy storage chain. For off-grid P2P systems centered on hydrogen-driven micro gas turbines, their technical and economic performance is highly sensitive to geographical location and renewable resource conditions, which significantly affect the system's installed capacity, seasonal hydrogen storage demand, and levelized cost of electricity. In the three cases of Palermo, Frankfurt, and Newcastle, the levelized cost of electricity is 0.86, 1.26, and 1.50 € per kWh, respectively, while the round-trip efficiency is approximately 15.7%, a final assessment of the system hybridised with battery storage shows a 20% levelized cost of electricity reduction and a 10% higher round-trip efficiency.^123^ In larger-scale scenarios of wind power absorption, the power-to-H_2_-to-power system using hydrogen-blended gas turbines can achieve flexible energy conversion and hydrogen self-sufficiency, while demonstrating good economic and environmental benefits. Its levelized cost of electricity and carbon emissions can reach 0.0537 € per kWh and 0.29 kg CO_2_-eq per kWh, respectively, and a hydrogen blending ratio of approximately 31% is considered to be the optimal threshold for achieving a balance between economic performance and emission reduction benefits,^124^ as shown in Fig. 7. More broadly, the SOFC–gas turbine hybrid cycle has the potential to achieve higher system efficiency. For example, the overall efficiency of the ammonia fuel inverted gas turbine–SOFC hybrid system can reach 68.45%, significantly higher than the operating level of a single SOFC. However, such efficiency improvement relies on more complex system integration and control, including heat exchanger layout optimization, dual turbine configuration, and air bypass regulation under varying operating conditions, and thus faces higher complexity in thermal management and dynamic operation.^125^ From an economic perspective, multi-objective optimization can be applied to determine key parameters such as compression ratio and pressure distribution, thereby balancing efficiency and cost.^126^ Technology rankings based on multi-criteria decision-making also support “hydrogen-blended natural gas” as a transitional pathway, providing a framework for investment decisions across different power ratings.^127^ Environmental assessments must simultaneously account for upstream hydrogen production pathways and NO_*x*_ control to avoid emission shifting.^128,129^ Specifically, although hydrogen use in gas turbines can reduce direct tailpipe CO_2_ emissions, lifecycle emissions may increase if the hydrogen is produced from carbon-intensive pathways, such as fossil-based reforming or electrolysis powered by high-carbon electricity. Additional energy demand for hydrogen compression, liquefaction, storage, and transport may further increase upstream emissions, while inadequate NO_*x*_ mitigation may shift the environmental burden from carbon emissions to air-pollutant emissions during combustion. Furthermore, the latest research has explored the advantages of hydrogen turbine engines, which offer zero carbon dioxide emissions and high energy density. However, they are constrained by the supply of green hydrogen, infrastructure, storage, combustion, leakage corrosion, and lifespan degradation. Therefore, it is necessary to develop micro-hybrid lean combustion, staged combustion, and source term estimation.^130–132^ Hydrogen-fueled operation may accelerate degradation of hot-section components, highlighting the need for health monitoring and life prediction models validated under variable operating conditions and long-term operation.^133^ Related studies have proposed design, operation and control methods for hydrogen-based hybrid fuel MGT systems by combining thermodynamic modeling. Among them, the cyclic regeneration structure is significantly superior to the simple cyclic structure in terms of fuel efficiency and waste heat recovery. On the other hand, integrating hydrogen into the CAES-HAT system can reduce emissions and improve system performance. The hydrogen fuel system performs best in thermodynamic indicators, but has higher cost rates and unit exergy costs, and requires multi-objective optimization to balance energy, economic performance and operational flexibility.^134,135^ The fuel system architecture proposed for the hydrogen-driven gas turbine engine, and it is pointed out that the selection and arrangement of components within the hydrogen fuel system remain key areas that require further research.^136^

In summary, hydrogen gas turbines are a key power regeneration technology in the electro-hydro coupling system that connects hydrogen production, storage, and power supply. However, hydrogen gas turbines still face challenges such as combustion stability, flashback, NO_*x*_ emissions, material corrosion, and lifespan degradation. Therefore, technologies such as micro-hybrid combustion, staged combustion, selective catalytic reduction, plasma-assisted ignition, source term estimation, and health monitoring need to be developed. The mixture of hydrogen and natural gas can balance fuel supply feasibility, compatibility with existing infrastructure, and gas turbine performance, while the mixture of hydrogen and ammonia can leverage the advantages of easy storage and transportation of ammonia and improve the low flame speed and ignition stability of ammonia combustion. Additionally, hydrogen-based gas turbines coupled with renewable electricity, hydrogen storage, and micro gas turbines systems can enhance energy conversion flexibility, emission reduction potential, and system efficiency, but their economic performance, full life cycle emissions, fuel system architecture, and model optimization still require further research.

### 4.3. Comprehensive utilization pathways of hydrogen in chemical, metallurgical, and transportation sectors

Within the electro-hydrogen synergy framework, hydrogen application pathways in the chemical, metallurgical, and transportation sectors highlight the pivotal role of power-to-X (PtX) in advancing cross-sectoral coupling, long-duration energy storage, and deep decarbonization. Table 2 provides a systematic summary of the representative hydrogen application pathways in these sectors, along with their major functions, principal advantages, and current challenges.

**Table 2:** Comparative overview of hydrogen utilization pathways in chemical, metallurgical, and transportation sectors

Sector	Chemical sector	Metallurgical sector	Transportation sector
Representative pathway	Green ammonia, green methanol, hydrogen-mediated power-to-X routes	Hydrogen-based direct reduction of iron and low-carbon high-temperature industrial heating	Fuel cell vehicles, hydrogen supply for heavy-duty transport, maritime, and aviation hydrogen pathways
Role of hydrogen	Hydrogen serves as a feedstock, energy carrier, and bridge between renewable electricity and chemical production	Hydrogen acts as a reducing agent and low-carbon fuel substitute in hard-to-abate industrial processes	Hydrogen serves as a zero-tailpipe-emission fuel and an energy carrier for long-range/high-load mobility
Major advantages	Enables conversion of intermittent renewable electricity into transportable chemical products; supports decarbonization of ammonia/methanol production; facilitates long-duration energy storage in chemical form	Offers deep decarbonization potential for steelmaking and other high-emission industries; can substantially reduce fossil-fuel dependence	High energy density, suitability for heavy-duty and long-range applications, strong potential for decarbonizing difficult-to-electrify transport modes
Key challenges	High production cost, sensitivity to renewable power fluctuation, limited large-scale validation of energy efficiency and techno-economics, additional losses during reconversion and purification	High hydrogen demand, process integration complexity, thermal management challenges, high infrastructure retrofitting cost, insufficient dynamic coupling with industrial load and supply variability	High fuel cost, insufficient refueling/storage infrastructure, safety constraints, onboard storage limitations, and limited empirical validation in complex real-world scenarios

In the chemical sector, green hydrogen facilitates the production of derivatives such as green ammonia and green methanol, thereby converting renewable electricity into chemical carriers amenable to storage and transportation. Research indicates that ammonia can serve as an efficient hydrogen carrier, alleviating storage and transport challenges. Emerging pathways, such as light-driven nitrogen fixation, also offer potential for low-carbon synthesis. However, these remain predominantly at the proof-of-concept or pilot scale, with insufficient validation of energy efficiency and economic viability at a commercial scale.^137,138^ Building on this foundation, ref. 139 and 140 indicate that integrating solar-driven electrolysis with CO_2_ capture and utilization for methanol production can substantially reduce lifecycle emissions, since renewable-based green hydrogen production emits less than 2 kg CO_2_ per kg H_2_, compared with 17–38 kg CO_2_ per kg H_2_ for hydrocarbon-based hydrogen production. Hydrogen's potential in facilitating the integration of intermittent renewable energies into the energy grid, thereby decarbonizing sectors that are challenging to electrify. Concurrently, ref. 141 and 142 demonstrate that reconverting ammonia or methanol derivatives to hydrogen incurs energy losses, and purification technologies currently struggle to simultaneously achieve high efficiency and chemical-grade purity, thereby constraining overall industrial chain efficiency.

In the metallurgical sector, hydrogen serves as a reducing agent, offering a deep decarbonization pathway for energy-intensive industries such as steelmaking. The anticipated improvements in electrolyzer efficiency and cost reductions in solar and wind technologies could further decrease the cost of hydrogen to $3.46 per kg and the total net present cost by over 25%, demonstrating the technical, economic, and policy viability of such systems integration for industrial decarbonization.^143^ However, challenges persist in process integration and operational stability for high-temperature direct reduction and full-process thermal management, cost assessments often fail to dynamically couple with specific production lines, load fluctuations, and hydrogen supply constraints. In heavy-duty sectors such as mining, hydrogen-powered equipment offers emission reduction potentials of 60–95%, although retrofitting costs and infrastructure gaps pose significant barriers to large-scale deployment.^144^ Despite a declining cost trajectory for green hydrogen, electrolyzer efficiency, operational lifespan, and capital expenditure remain critical constraints. There is an urgent need for integrated assessments that link hydrogen production parameters with downstream industrial demand, operational strategies, and carbon pricing scenarios.^145,146^

In the field of transportation, hydrogen is gradually becoming an important carrier for promoting the transformation of low-carbon travel and the reconfiguration of the transportation energy system. The refined energy management strategies for complex operating conditions provide new technical paths for reducing hydrogen consumption, improving power response, and optimizing the overall system efficiency. Regarding hydrogen storage and transportation, compressed gaseous hydrogen is limited by its relatively low volumetric energy density. Although cryogenic liquid hydrogen can increase the hydrogen storage density, the liquefaction process has high energy consumption and poses higher requirements for insulation and thermal management. Solid-state hydrogen storage still faces issues such as larger system mass, limited hydrogen absorption and release kinetics, and insufficient cycle stability.^147^ Additionally, ref. 148 further points out that compressed hydrogen, although relatively low in cost, has a large storage volume, which is not conducive to the utilization of vehicle space. The liquid hydrogen, although having a high energy density, requires additional energy consumption and more complex thermal management. Pipeline transportation is suitable for long-distance and large-scale transportation, but its initial infrastructure investment is high, and the construction period is long. Hydrogen applications, particularly in fuel-cell transport and hydrogen-mediated multi-energy systems, can deliver zero direct tailpipe emissions at the point of use while enabling cross-sectoral coupling among the power, transport, industrial, and building sectors through electricity–hydrogen conversion and reconversion pathways, thereby enhancing renewable energy utilization.^149,150^ Nevertheless, methods for large-scale power generation (more than 100 MW) using a combination of renewable energy sources, water electrolysis, and hydrogen fuel cells still need to be studied in depth.^151^

In summary, current research generally lacks large-scale, system-level validation of economic viability and operational stability. Transportation-related energy storage and management strategies exhibit insufficient adaptability in real-world environments, and material-related bottlenecks remain unresolved. Future efforts must prioritize cross-scale integrated validation within electricity-hydrogen coupling systems, alongside full-chain techno-economic and life-cycle assessments. Such endeavors are essential to establishing replicable system-level solutions and closed-loop engineering data for representative scenarios in the chemical, metallurgical, and transportation sectors, thereby supporting a scalable low-carbon transition.

## 5. Architecture and control of electricity-hydrogen coupling systems: integration technologies and multi-timescale optimization

### 5.1. System architecture design for electricity-hydrogen coupling: adaptation to fluctuating power sources and efficiency optimization

Water electrolysis constitutes the critical interface for converting renewable electricity into green hydrogen, with technological advancements increasingly oriented toward high efficiency, low cost, and enhanced adaptability to fluctuating power sources. Alkaline water electrolysis, leveraging its technological maturity and cost advantages, remains a prominent option for large-scale deployment, with research efforts concentrated on improving its dynamic response capabilities. Existing studies have evaluated the impact of wind power fluctuations on industrial-scale AWE systems through dynamic modeling, proposing operational strategies to mitigate overheating and enhance energy efficiency.^152^ Furthermore, the design and experimental validation of solar-driven multi-stage alkaline electrolysis units demonstrate favorable scalability and adaptability to power fluctuations, with higher energy efficiency observed under single-stack low-load operation.^153^ However, such investigations are generally confined to specific scenarios or short-term operational regimes, lacking in-depth analysis of material degradation and system lifespan for AWE under prolonged, wide-range power fluctuations.

**Fig. 8:**
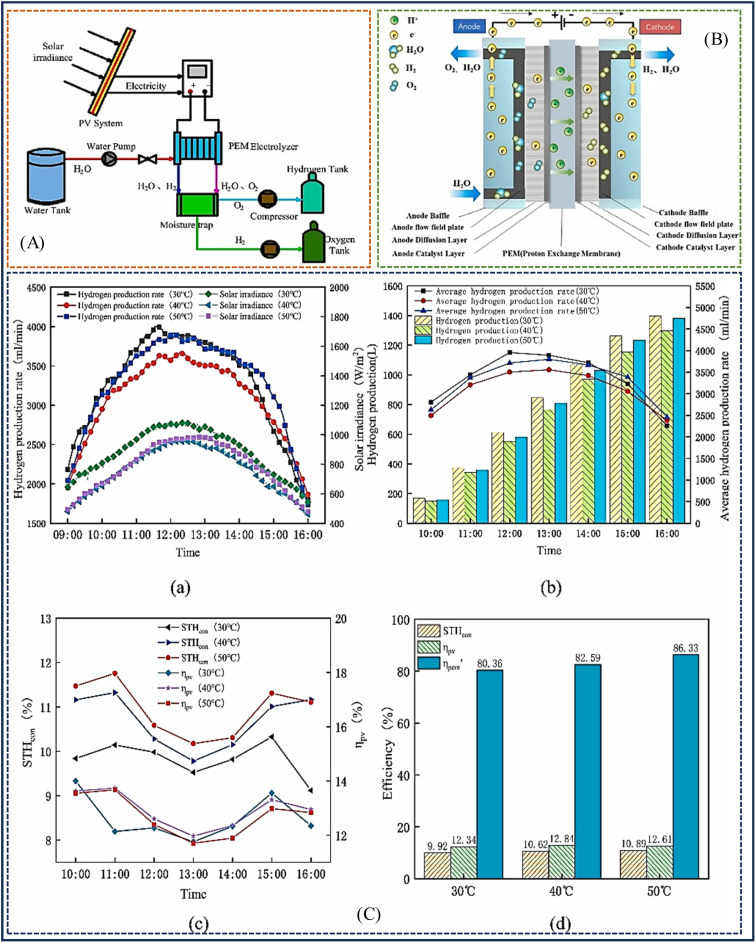
(A) Schematic of a PV direct-coupled system for PEM hydrogen production; (B) structure and schematic of the PEM electrolyzer; (C) changes in hydrogen production rate and efficiency of the system under different inlet water temperatures: (a) instantaneous hydrogen production rate change; (b) hydrogen production quantity and hourly average hydrogen production rate change; (c) changes in PV electric efficiency and STH con under different water inlet temperatures; (d) changes in total efficiency under different water inlet temperatures. Adapted from ref. 155 with permission from Elsevier: Xi Weng, Zhen Jia, Wei He, *et al.*, *Renewable Energy*, 2026, **256**, Part D, 124 133. Copyright © 2025 Elsevier Ltd.

Compared with AWE, SOEC offers the theoretical efficiency under high-temperature operating conditions. Research has optimized their start-up and steady-state transition processes using one-dimensional dynamic models, underscoring the critical role of thermal management in achieving efficient and responsive performance.^154^ Nevertheless, the fundamental bottleneck to SOEC commercialization remains the chemical stability and degradation mechanisms of key materials under prolonged high-current-density operation, with a gap between fundamental materials science and engineering-scale implementation. The PEM has garnered significant attention due to its rapid response capabilities and high efficiency. Experimental platforms for photovoltaic direct-coupling reveal that system efficiency and hydrogen production rate are highly sensitive to solar irradiance and the electrical characteristics of PV modules,^155^ as illustrated in Fig. 8. Capacity optimization studies for wind-PV-PEM hybrid systems further indicate that PEM capital cost is the primary component of the levelized cost of hydrogen.^156^

In terms of adaptability to power fluctuations, a grid-integrated PV-powered PEM electrolysis system was proposed, in which grid-connected operation increased the conversion efficiency to 95.6%, compared with 81.2% in stand-alone mode, while maintaining total harmonic distortion below 3.76%, thereby improving power quality and supporting stable electrolyzer operation under variable solar irradiance.^157^ While off-grid/microgrid mode relies on internal system optimization, using integrated battery energy storage^158,159^ or adopting intelligent control strategies^160^ to smooth out power fluctuations, improve operational stability, and energy efficiency. To address the issues caused by intermittent power, ref. 161 proposes a hydrogen production system consisting of solar panels, batteries, commercial PEM electrolyzers, and electrical control strategies, which can improve efficiency and reduce the risk of electrolyzer damage, making the process more sustainable and reliable. On the whole, AWE urgently needs to complete long-term dynamic durability data, SOEC needs to establish a cross-scale collaborative path between material degradation mechanisms and engineering scaling, and PEM research is relatively weak in the analysis of the correlation between full life cycle costs and material degradation in real scenarios. Therefore, research on electrolysis water hydrogen production technology needs to further strengthen long-term reliability, empirical studies, material-system cross-scale collaborative design, and full-chain economic assessment based on real market data to support its large-scale commercial application.

### 5.2. Electro-hydro coupling system integration technology: coupling modeling from microgrid to regional energy system

**Fig. 9:**
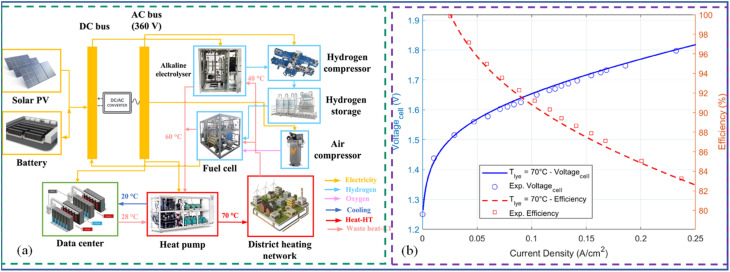
(a) Schematic diagram of a designed green data center. (b) Comparison between simulated and experimental data for the Phoebus electrolyzer. Adapted from ref. 165 with permission from Elsevier: Juan Jose Garcia Pabon, *et al.*, *International Journal of Hydrogen Energy*, 2025, **196**, 152 557. Copyright © 2025 Hydrogen Energy Publications LLC. Published by Elsevier Ltd.

The integration of electrolysis for hydrogen production, hydrogen storage, hydrogen power generation, renewable power generation, traditional power grids, and diverse loads in a coordinated manner is an important path for building a high proportion of renewable energy systems. The system scale can extend from green data centers and industrial park microgrids to regional grid peak shaving and long-term energy storage.^162–164^ Ref. 165 constructed a green data center model integrating photovoltaics, batteries, alkaline electrolyzers, PEM fuel cells, and waste heat recovery. The waste heat recovery of the hydrogen system can increase the effective energy utilization efficiency to 82.3%, as shown in Fig. 9. However, such models often rely on idealized thermal coupling assumptions and do not fully assess the impact of the efficiency decay, maintenance costs, and availability changes of key equipment under long-term variable operating conditions on the system's economic performance. Further, the integrated energy system operation model considering carbon trading and demand response can achieve a synergistic improvement in economic and emission reduction under certain conditions,^166^ but the resilience analysis of real risks such as policy fluctuations, incomplete markets, and raw material supply disruptions is still relatively weak.

Current research generally focuses on multi-objective optimization, integrating artificial intelligence and advanced algorithms to solve capacity allocation and real-time scheduling problems, aiming to balance economic efficiency, environmental benefits, and technical reliability.^167,168^ Ref. 169 proposed a multi-objective capacity configuration optimization model for a coupled system integrating wind power, photovoltaic power, electrolyzers, hydrogen storage equipment, and hydrogen fuel cells. However, such models usually rely on determined or simplified uncertain parameters and do not adequately depict the long-term randomness of wind and solar power generation and load demand. Ref. 170 used an improved deep reinforcement learning algorithm to schedule a cold, heat, electricity, hydrogen, and fuel cell hybrid system, achieving cost reduction. However, its performance is dependent on the quality of training data and model accuracy, and the decision reliability, interpretability, engineering portability, and cost-benefit closed-loop verification in practical engineering still need to be strengthened.

In summary, although research on integrated electro-hydrogen systems has made progress in model development and algorithmic innovation, long-term dynamic risks such as equipment aging and market volatility have not been fully accounted for, and high-dimensional uncertainties have been addressed in a simplified manner. System reliability testing of intelligent algorithms in real-world scenarios lacks systematic approaches. Therefore, it is necessary to rely on long-term empirical data to develop a model system capable of coupling uncertainties across multiple time scales as well as equipment aging, and to conduct coordinated technical, economic, and safety validation.

### 5.3. Multi-timescale operational control of electricity-hydrogen coupling systems: dynamic response and dispatch optimization

The operational control of electricity-hydrogen coupling systems encompasses multiple timescales ranging from second-level dynamic responses to seasonal dispatch, necessitating the coordination of device-level rapid control and system-level optimal dispatch to ensure stable, efficient, and economical operation under severe power fluctuations.

At the seconds-to-minutes timescale, the objective of wide-range dynamic response control is to mitigate power quality issues arising from renewable power step changes, start-stop operations, and disturbances, thereby ensuring stable and efficient operation of electrolyzers and power electronic equipment under fluctuating conditions. To address challenges associated with DC bus voltage fluctuations and high current ripple in direct wind-AWE coupling systems, interleaved parallel virtual DC motor control enhances transient stability by introducing virtual inertia and damping.^171^ For off-grid offshore wind hydrogen production scenarios, a time-domain coupled model incorporating PEM electrolyzer stacks, together with coordinated start-stop control strategies, reduces unplanned shutdowns by 62%, thereby improving equipment lifespan and hydrogen production levels.^172^ However, the above studies are predominantly based on averaged or simplified models, exhibiting insufficient characterization of sub-second processes such as internal electrochemical dynamics and thermal inertia within electrolyzers. Consequently, their robustness under extreme fluctuations, fault disturbances, and communication delays remains inadequately validated.^173^

**Fig. 10:**
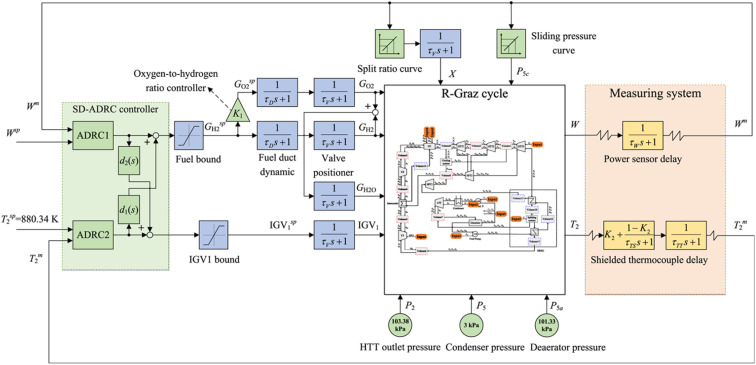
Schematic diagram of the control system of the R-Graz cycle. Adapted from ref. 174 with permission from Elsevier: Chengbo Dai, Shida Yu, Shaojie Liu, *et al.*, *International Journal of Hydrogen Energy*, 2025, **188**, 152 113. Copyright © 2025 Hydrogen Energy Publications LLC. Published by Elsevier Ltd.

**Fig. 11:**
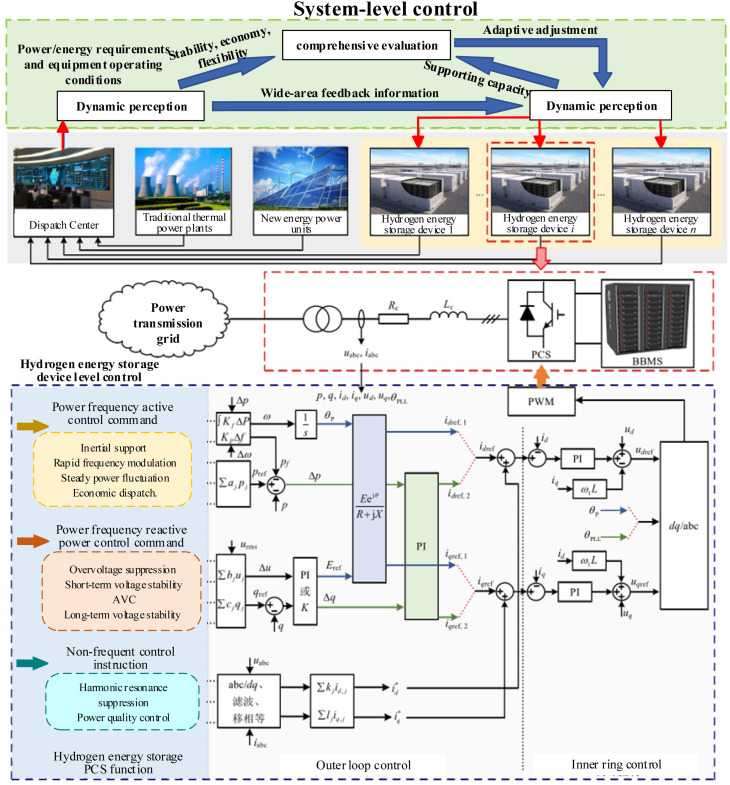
The multi-time-scale control concept model of the electro-hydrogen system.

To achieve efficient and flexible power generation from hydrogen, dynamic modeling and control of novel power cycles have emerged as a burgeoning research focus. Ref. 174 presents the dynamic modeling of an improved Graz cycle and the design of an active disturbance rejection decoupling controller, enabling rapid tracking of grid power dispatch signals and operation as a flexible peak-shaving power source. Fig. 10 illustrates the schematic diagram of the R-Graz cycle control system. While this research addresses the limitations of unidirectional power-to-hydrogen control, the complexity of such models and controllers poses potential challenges in terms of reliability and maintenance costs for practical engineering implementation. Furthermore, dynamic delays arising from the coupling between fuel supply systems and combustion processes require further integration into the control framework. Therefore, the construction of digital twin platforms integrating multi-physics fields and finely resolved multi-timescale models, coupled with long-term empirical validation under realistic fluctuating scenarios, constitutes a critical direction for enhancing the engineering credibility of control strategies. Fig. 11 presents a conceptual model of multi-timescale regulation for electricity-hydrogen systems, encompassing two hierarchical levels: system-level control and hydrogen energy storage device-level control.

At longer timescales ranging from hours to days and seasons, multi-timescale optimal dispatch aims to integrate seasonal energy balancing, day-ahead/intra-day scheduling, and real-time control execution, thereby achieving overall optimality in terms of economic efficiency, reliability, and low-carbon performance. Research on inter-seasonal energy storage has proposed two different cross-seasonal hydrogen storage operation strategies and developed multi-scale optimization frameworks spanning annual, daily, and hourly resolutions. The multi-time scale optimization reduced the penalty cost in the summer cross-seasonal hydrogen storage stage by 93.36%.^175^ Capacity planning studies integrating nuclear energy with hydrogen systems further elucidate the complementary value of stable power sources and long-duration energy storage,^176,177^ confirming the role of hydrogen in addressing annual/seasonal energy imbalances. However, such planning models often substantially simplify the actual operational constraints and flexibility limitations of key equipment.

At the short-term and medium-term scheduling level from the current to the near future, the research focuses on optimizing operational economy. Ref. 178 has formulated the optimal operation rules for the hydropower-hydrogen-solar and wind hybrid system, considering the linkage of the electricity-hydro market. Ref. 179 and 180 introduce the stepped carbon trading mechanism in the scheduling of the electricity-heat-gas-hydro integrated energy system to optimize the multi-energy flow coordination. These research mechanisms are dependent on preset and often idealized market electricity prices and policy parameters. The adaptability analysis for the actual market incompleteness and policy sudden changes is weak. At the real-time level, methods such as fuzzy logic and model predictive control can achieve the second/minute-level power allocation of hydrogen-electric mixed energy storage ref. 181 and 182. Although the control effect is significant, these strategies rely on high-precision models and an ideal communication environment. Their robustness and scalability under real disturbances such as equipment failures and communication delays have not been fully verified.

In summary, although existing studies have established multi-scale optimization frameworks covering the range from seasonal to real-time, they have failed to adequately consider the resilience against inter-annual climate variability, electricity price fluctuations, and policy uncertainties in long-term scheduling. The medium-term models have not accurately reflected the actual operational limitations of key equipment and market flaws, and there is a lack of systematic verification of the robustness and scalability of short-term control strategies under actual disturbances such as equipment failures and communication delays. It is necessary to establish a unified scheduling theory and verification platform. This platform should be capable of integrating uncertainties across different time scales and depths, thereby enabling the large-scale and reliable operation of the power-hydrogen coupling system in complex power market environments and under conditions of high volatility in power sources.

## 6. Application value of the electro-hydrogen synergy system: renewable energy consumption, auxiliary services, and low-carbon transformation

### 6.1. Large-scale renewable energy consumption and long-term energy storage technology of the electro-hydrogen synergy system

In a high proportion of renewable energy power systems, the intermittent, fluctuating, and seasonal imbalances have intensified. Hydrogen, with its high energy density, long storage period, and the ability to be stored and transported across seasons and regions, is regarded as a key path to achieve large-scale renewable energy consumption and long-term energy storage.^183–185^ Its system value is primarily reflected in multi-sector coupling, hydrogen can connect electricity with demand sides such as transportation, industry, and building heating,^186–188^ and collaborate with heat networks^189,190^ and natural gas networks^191,192^ to enhance the efficiency and resilience of the integrated energy system. However, the existing coupling studies are based on theoretical modeling and static efficiency comparisons. And fail to fully consider the differences in the dynamic responses of different entities, the costs of infrastructure collaborative transformation, and the operational benefits across entities.

**Fig. 12:**
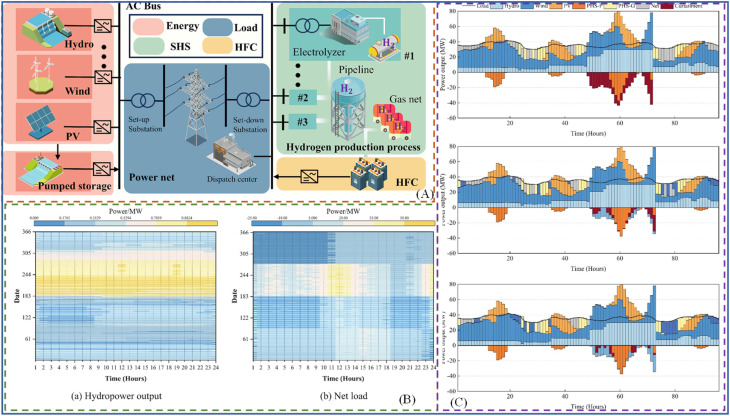
(A) Hydropower-hydrogen coupled multi-energy system. (B) Hydropower output and net load throughout the year. (C) Hourly power balance results of typical daily energy output under three scenarios in the power system model over four seasons. Adapted from ref. 195 with permission from Elsevier: Zhipeng Yang, Xiaobo Zheng, Shuaihui Sun, *et al.*, *Energy Conversion and Management*, 2026, **348**, Part C, 120 768. Copyright © 2025 Elsevier Ltd.

From a time scale perspective, hydrogen storage can make up for the insufficient duration of battery storage and is an important means to alleviate the contradiction between supply and demand over multiple days and seasons.^193^ Relevant studies have pointed out that hydrogen has advantages in energy density and long-term storage,^194^ and through model verification, it can reduce wind and solar energy waste, alleviate seasonal imbalances,^195^ as shown in Fig. 12. However, most of the work is based on idealized efficiency and cost parameters and lacks systematic evaluations of cross-seasonal hydrogen storage energy loss, equipment idling aging, and the economic performance throughout the entire life cycle.

From a spatial scale perspective, hydrogen can be transported through pipelines, ships, or roads to achieve cross-regional energy optimization allocation, converting renewable electricity from resource-rich areas into hydrogen and transporting it to load centers.^196^ Regarding the shared energy storage and hydrogen logistics, existing research has proposed shared frameworks based on game theory, low-carbon economic scheduling, and multi-layer hydrogen storage schemes,^197,198^ and some work has focused on route and distribution optimization for point-to-point green hydrogen production supply chains.^199^ Meanwhile, a novel liquid-hydrogen-based solar energy system was simulated to meet electricity demand, the system's total and storage round-trip efficiency (RTE) are 53.3% and 38.1%, respectively, primarily due to significant exergy losses in the electrolyzer (34.9%), hydrogen liquefaction (23%) and fuel cell (38%).^200^

**Fig. 13:**
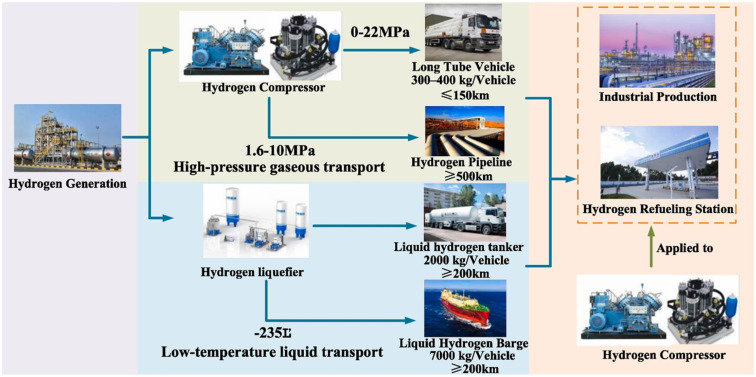
Diagram of different modes of transportation. Adapted from ref. 205 with permission from Elsevier: S. Zhang, Y. Teng, H. Kong, *et al.*, *Renewable and Sustainable Energy Reviews*, 2025, **223**, 116 009. Copyright © 2025 Elsevier Ltd.

At the system-level planning and scheduling, research is dedicated to enhancing overall economic efficiency and low-carbonity through collaborative optimization. Literature^201^ proposed a forward-looking scheduling model for solid-state transportation mode based on magnesium-based hydrogen transport vehicles (MHTV), and verified its effectiveness through simulation. Subsequently, literature^202^ introduced a phased collaborative planning model for the power-hydrogen-transportation coupling network, reducing total costs and carbon emissions by coordinating multiple networks. Literature^203^ proposed an optimal coordination configuration method for power and hydrogen storage, using the energy hub framework to promote the utilization of renewable energy. However, the models are generally complex and rely heavily on deterministic predictions of demand, costs, and policies, and the robustness and feasibility in the face of multiple uncertainties still need to be verified. Supply chain review studies further compared renewable hydrogen production systems, storage, and transportation routes and environmental impacts, indicating that material-based transportation has higher safety potential and emphasizing the direction for improving control accuracy and safety in the compression and transportation stages.^204^ Literature^205^ improved the performance of hydrogen compressors through integrated variable frequency drives and outlined various transportation methods, as shown in Fig. 13, proposing the uniqueness and adaptability of each transportation method.

Large-scale and low-cost storage is the core for realizing the long-term value of hydrogen energy storage. Underground hydrogen storage is considered the most promising option as it has a large capacity and low cost. Literature^206,207^ discussed the technical and economic feasibility of scenarios such as depleted oil and gas reservoirs, but issues such as geological adaptability risk assessment, long-term injection and production-induced formation stability, microbial effects, and gas mixtures were not fully addressed. On the other hand, hydrogen is usually combined with battery energy storage to form a hybrid energy storage system. Literature^208^ explored that batteries are responsible for achieving smooth power regulation within seconds to hours, while the hydrogen system is responsible for storing excess power on daily, multi-day, and seasonal time scales. However, the proposed optimization planning and control strategies are mostly based on idealized models, and they are insufficient for real-time and robust verification in the face of uncertain energy and load conditions, as well as multi-time-scale coupling situations. Thus, it is necessary to conduct in-depth scientific research on geological risks of underground hydrogen storage, long-term dynamic process modeling, coordinated control of hybrid energy storage, and optimization configuration and standardized evaluation at different time scales.

### 6.2. Auxiliary services of power system: peak load regulation, frequency regulation, and reserve capacity

Hydrogen can participate in peak load regulation, frequency regulation, and reserve capacity through equipment such as electrolyzers, hydrogen storage systems, and fuel cells, achieving coordinated optimization with renewable energy.

In terms of peak load regulation, hydrogen mainly absorbs excess renewable energy through electrolyzers to alleviate the problem of wind and solar power curtailment. Hydrogen production from offshore wind and integrated energy systems, through multi-level market optimization, has improved economic efficiency and power consumption rate, but their models usually ignore actual engineering limitations and the dynamic complexity of the power market.^209,210^ The control strategies for off-grid and wind-hydrogen-storage systems use multi-objective rolling optimization and variational mode decomposition, reducing the start-stop of electrolyzers and enhancing peak load regulation capacity, but their methods are computationally complex and difficult to directly extend to large-scale grid applications.^211,212^ The research on the application of hydrogen storage in large-scale grids has confirmed its value for long-duration and seasonal balancing. Ref. 213 examined the potential of large-scale thermal energy storage and hydrogen as seasonal energy storage technologies in achieving energy self-sufficiency in regions with renewable energy. Ref. 214 clarified the differences between short-term and long-term storage in reducing the cost of fixed photovoltaic power generation. When a 100% photovoltaic power supply system meets the average daily load demand of 22.04 megawatt-hours, the confirmed kilowatt-hour premium obtained is 5.42. However, to achieve the best system cost, 44.81 megawatt-hours of batteries, 684 kilowatts of electrolyzers, and 540 kilowatts of fuel cells need to be installed. In addition, hydrogen-battery hybrid energy storage can complement power and capacity characteristics, enhancing the system's peak load resilience and reliability.^215^ However, overall, the existing research on peak load regulation is lacking in model practicality, economic analysis for large-scale applications, and design of market-based coordination mechanisms.

**Fig. 14:**
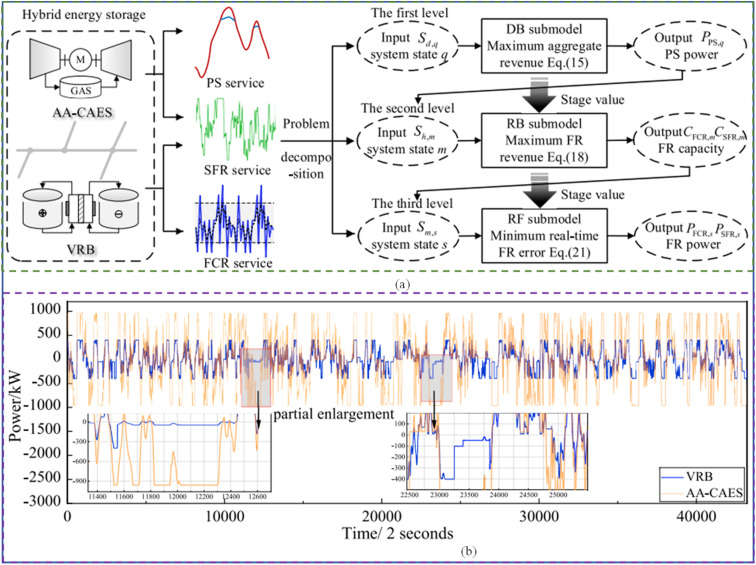
(a) The optimal framework for HESS participation in multiple ancillary service markets. (b) Daily dispatch curve of HESS. Adapted from ref. 218 with permission from Elsevier: Yan Yao, Ye He, Hongbin Wu, *et al.*, *Journal of Energy Storage*, 2025, **105**, 114 677. Copyright © 2024 Elsevier Ltd.

In terms of frequency regulation, the hydrogen system can participate in frequency control by its rapid power regulation capability, forming a complement to short-term energy storage.^216,217^ Based on this, ref. 218 proposed an optimal scheduling strategy, which applied a hybrid energy storage system to DG power generation and the multi-support service market. As a result, the comprehensive revenue increased by 4.87%, and the auxiliary service revenue rose by 15.2%, as shown in Fig. 14. Ref. 219 a resilience-oriented energy management scheme for an electricity-hydrogen DCµG considering participation of the DCµG in the automatic frequency restoration reserve market, participating in the automatic frequency recovery reserve market can increase profits by approximately 30.5%, while adopting a resilience-oriented scheduling strategy can reduce the cost of power curtailment by about 16.72% during emergency periods. There are also works using game theory and model predictive control to optimize the frequency regulation strategy of the electro-hydrogen microgrid.^220,221^ However, there is still a need for further in-depth research in terms of dynamic modeling accuracy, the precise matching of the frequency regulation requirements for high proportion renewable energy systems, and the robust online control that can handle various disturbances and different grid structures.

In terms of backup and flexible ramping, electrolytic cells, hydrogen refueling stations, and the electro-hydro-transportation coupled resources can provide backup capacity and resilience support. Relevant studies have shown that participating in the flexible ramping market can enhance revenue and consumption.^222,223^ Incorporating hydrogen refueling stations into the virtual power plant can enhance backup capacity through data-driven and stochastic optimization ref. 224 and 225. Meanwhile, the grid-hydro-vehicle collaboration in extreme event scenarios can reduce load reduction and improve backup reliability,^226^ and risk management methods are used to depict the impact of prices and uncertainties ref. 227 and 228. However, there are still issues such as insufficient implementation of trading rules and incentive policies, limited generalization ability of data-dependent methods under incomplete information, and insufficient comprehensive decision-making frameworks for multi-risk extreme scenarios.

The advantages of hydrogen energy in power system auxiliary services such as peak shaving, frequency regulation, and backup are obvious. However, current research lacks empirical verification under real operating environments and market conditions, and does not fully consider the dynamic performance decay of equipment, the full life cycle cost and benefit risks. The need to consider the collaborative mechanism between electricity, hydrogen energy and the transportation network is still in its infancy. It is necessary to establish refined technical and economic assessment and performance decay models, develop robust/distributed optimization and real-time control schemes for uncertainties. A market and regulatory framework compatible with incentives should be constructed to fully release the system value of hydrogen energy auxiliary services.

### 6.3. Low-carbon transportation and industrial system construction: integrated pathways for hydrogen energy in multiple scenarios

Hydrogen energy possesses advantages such as zero emissions, high energy density, and cross-sectoral coupling in the deep decarbonization of transportation and industry. In the transportation sector, fuel cell vehicles are suitable for sectors that are difficult to electrify, such as heavy trucks and ships. Research indicates that integrating renewable energy sources like photovoltaics into hydrogen supply infrastructure can reduce emissions throughout the life cycle, but it is limited by photovoltaic efficiency and hydrogen production costs.^229^ New models, such as floating hydrogen production and refueling barges for ships, provide feasible demonstrations for hydrogen supply to ships, but their scale and universality still need to be verified.^230^ On the sector coupling for maritime decarbonization, literature^231^ emphasizes the importance of energy and industrial linkage, while also suggesting that social and technological factors, such as resource competition, may be constraints. From a policy and strategic perspective, studies suggest that China's transportation sector needs to further reduce the cost of fuel cells and improve the regulatory system to accelerate industrialization,^232^ but the micro-market incentives and industrial chain synergy mechanisms still lack operational design. The review on freight decarbonization also reflects the insufficiency of empirical data and quantitative evidence for emission reduction.^233^

In the industrial sector, the substitution of fossil fuels with hydrogen in industries that are difficult to decarbonize, such as steel and cement, is an important path for achieving deep emission reduction. Relevant reviews emphasize the urgency of low-cost and low-carbon hydrogen supply, but social and technological factors such as investment risks, political and social acceptance are often underestimated.^234^ The comparisons of different hydrogen production routes reveal trade-offs between environmental benefits, water resource consumption, and energy efficiency of green hydrogen, and there are also trade-offs between fossil hydrogen production and costs and carbon emissions, but there is still a lack of comprehensive selection and system integration research for specific industrial scenarios. Meanwhile, collaborative research on CCUS and hydrogen production provides indirect support for low-carbon hydrogen supply.^235^ Hydrogen supply pathways mediated by carriers such as ammonia and key catalytic technologies are also developing, but catalyst costs, lifespan, and dynamic performance may still be bottlenecks for large-scale implementation.^236^

Hydrogen energy has significant advantages in building a low-carbon transportation system and promoting industrial decarbonization. However, existing research mostly focuses on the description of technical paths or macro-strategic analysis, lacking technical-economic quantitative data and empirical evidence for complex actual scenarios. Currently, there has not been a systematic coupling design, conflict coordination mechanism, and value distribution mechanism covering the transportation-industry-energy network. Regarding key scientific issues such as low-cost hydrogen electrolysis technology, the stability of key material supply, and the key capabilities for large-scale storage and transportation, it is urgent to build a multi-dimensional unified assessment system and a refined modeling framework to provide systematic theoretical support for accelerating the application of the electro-hydrogen collaborative system in the low-carbon transformation.

## 7. Development suggestions and future perspectives for electro-hydrogen synergistic systems

### 7.1. Accelerate breakthroughs in key materials and core technologies across the hydrogen value chain

For the development of the hydrogen production process, efforts should be made to accelerate the research and development of key materials and core technologies. Currently, high-performance electrolyzers are dependent on precious metal catalysts and highly stable membrane materials. The non-precious metal systems still have deficiencies in dynamic response, durability, and adaptability to operating conditions. The high-temperature hydrogen production route also faces challenges such as material degradation and long-term reliability. These are the core issues for achieving low-cost and large-scale hydrogen production. By promoting breakthroughs in key technologies such as low-precious-metal and non-precious-metal catalysts, high-stability membrane materials, and anti-degradation electrode structures, combined with means like materials genomics, nanotechnology, *in situ* sensing, online monitoring, and predictive maintenance, the efficiency, reliability, and predictability of the hydrogen production system can be effectively enhanced.

For hydrogen storage, transportation, and conversion utilization, diversified technological pathways should be developed according to application-specific requirements. High-pressure gaseous storage is limited by low volumetric energy density and high equipment costs; liquid hydrogen storage faces high liquefaction energy consumption and boil-off losses; solid-state storage must balance capacity, kinetics, and cycling stability; and liquid organic hydrogen carriers are constrained by dehydrogenation energy demand and overall system efficiency. In utilization, further improvements are needed in fuel-cell catalyst durability and life-cycle cost, lightweight and safe storage–transport systems, and hydrogen gas turbines with enhanced resistance to hydrogen embrittlement and NO_*x*_ emissions. Therefore, coordinated research on solid-state storage, low-temperature storage, and advanced liquid organic carriers should be strengthened. Multi-scale simulation and machine learning can accelerate material screening, structural design, and operating-state optimization, while material substitution, fuel adaptability, and health management technologies should be integrated to enhance the performance and operational intelligence of electro-hydrogen synergistic systems.

### 7.2. Improve the multi-time-scale dynamic coupling and collaborative control mechanism of the electro-hydrogen synergy system

For the development of the electro-hydrogen synergy system, it is necessary to further improve the system-level multi-time-scale dynamic coupling and collaborative control mechanism. The electro-hydrogen synergy system needs to achieve unified management among second-level disturbance response, minute-level power regulation, hour-level optimization scheduling, and seasonal-level energy balance. At the equipment control level, efforts should be made to strengthen the modeling of the electrochemical-thermodynamic rapid dynamic process of the electrolyzer, enhancing its robustness in the face of renewable energy fluctuations and extreme operating conditions. At the optimization scheduling level, long-term planning, medium-term scheduling, and short-term real-time control should be coordinated, and the adaptability to climate, load, electricity price, carbon price, policy parameters, as well as equipment failures, communication delays, and measurement noise, *etc.*, should be strengthened. At the multi-energy synergy level, real-time collaborative scheduling, safety boundary delineation, and risk propagation mechanism research for hydrogen energy, battery storage, heating networks, and natural gas networks should be enhanced.

In response to the contradiction between intermittent renewable energy and the stable operation of electrolyzers, it is necessary to promote refined collaboration among the power grid, energy storage, and hydrogen production systems, and improve the coordination mechanism of hydrogen as cross-seasonal and short-term energy storage. For centralized hydrogen production, distributed applications, and diversified load scenarios, the integrated design of system planning, capacity configuration, and operation control should be strengthened to enhance the flexibility, safety, and economy of the electro-hydrogen synergy system in multiple scenarios.

### 7.3. Promote the intelligent integration and collaborative control upgrade of the electro-hydrogen synergy system

The development of electro-hydrogen synergy systems requires further in-depth research on system integration and control toward high intelligence, digitalization, and deep coupling of multiple energy flows. Based on the coupling pathways among sources, grids, loads, energy storage, and hydrogen, future research should shift from single-point optimization strategies to system-level intelligent platform construction. By integrating digital twin technology, data-driven models, and high-precision mechanism models, a digital-intelligent coordination framework can be established to support real-time state perception, dynamic performance prediction, operational decision-making, and coordinated control of electro-hydrogen systems.

At the same time, the collaborative planning and operation of multiple energy flows, including electricity, hydrogen, thermal energy, and natural gas, should be further strengthened. Integrated models such as energy hubs can be used to describe cross-energy conversion, storage, and interaction mechanisms, while advanced control methods such as robust control and model predictive control can improve system stability, flexibility, and operational efficiency under fluctuating conditions and uncertain environments. In addition, future studies should pay more attention to demonstration validation, standardization, and life-cycle assessment. Through pilot-scale demonstrations, unified technical standards, and full-chain evaluations covering energy efficiency, carbon emissions, economy, safety, and environmental impacts, the practical applicability and scalability of electro-hydrogen synergy systems can be comprehensively improved.

## 8. Conclusions

In the context of the concurrent advancement of a high proportion of renewable energy integration and deep decarbonization requirements, the electro-hydrogen synergy system has become an important path for promoting the consumption of a high proportion of renewable energy, achieving energy regulation across different time scales, and driving the low-carbon transformation in multiple fields. The paper reviews the research progress of the P–H system in hydrogen production, hydrogen storage, system synergy, and commercialization, and focuses on analyzing the main bottlenecks in key materials, multi-time-scale control, full value chain integration, and standard and market mechanism construction. The study shows that hydrogen production technology is shifting from solely pursuing efficiency improvement to considering durability, economy, and system synergy under fluctuating conditions. Technologies such as PEMW, ALK, AEM, and SOEC each have their advantages, but still need continuous breakthroughs in low-cost material development, membrane electrode structure stability, *in situ* diagnosis at the stack level, and dynamic durability evaluation. The hydrogen storage path essentially involves a comprehensive trade-off between energy density, efficiency loss, safety, cost, and infrastructure compatibility. It needs to be optimized in collaboration with the operating pressure of the electrolyzer, purification and compression energy consumption, hydrogen quality constraints, and the fluctuation characteristics of renewable energy. At the system level, hydrogen should be transformed from a by-product to a flexible energy carrier, deeply participating in the multi-energy coupling system of electricity-hydrogen-heat-gas, and summarizing the architecture evolution, dynamic response control, and multi-time-scale optimization scheduling methods of the multi-energy coupling system in microgrids, industrial parks, and regional integrated energy systems, achieving cross-time-scale collaboration from millisecond-level grid support to seasonal energy balance.

In the future, as key materials continue to make breakthroughs and system modeling and control capabilities keep improving, research will focus more on durability assessment under fluctuating operating conditions, degradation perception control, life cycle technical and economic analysis, as well as the collaborative construction of safety standards, certification systems and market mechanisms. It will further explore the comprehensive value of the P–H system in energy transition and deep industrial decarbonization, providing support for the construction of a modern energy system that is clean, low-carbon, safe, efficient, flexible and resilient.

## Author contributions

Jie Wang: methodology, investigation, formal analysis, data curation, conceptualization, writing – review & editing. Bing Hu: conceptualization, supervision, methodology, writing – review & editing. LiJun Xu: conceptualization, methodology, validation. Xiaochao Fan: validation, data curation, investigation. Jiading Jiang: methodology, data curation, validation.

## Conflicts of interest

The authors declare that they have no known competing financial interests or personal relationships that could have appeared to influence the work reported in this paper.

## Data Availability

No new data were created or analysed in this study. As this is a review article, data sharing is not applicable to this work.
